# *Bacillus velezensis* Isolate X5 Stimulates the Resistance of Resistant and Susceptible Banana Varieties to Foc Through Different Mechanisms

**DOI:** 10.3390/jof11050379

**Published:** 2025-05-16

**Authors:** Yunlong Xu, Jun Wang, Guangxiang Tian, Changcong Liang, You Zhou, Lijia Guo, Yang Yang, Laying Yang

**Affiliations:** 1 National Key Laboratory for Tropical Crop Breeding, Key Laboratory of Integrated Pest Management on Tropical Crops, Ministry of Agriculture and Rural Affairs, National Collection of Microbial Resource for Fertilizer (Hainan), Collection of Tropical Agricultural Microbial Resource in Hainan Province, Environment and Plant Protection Institute, Chinese Academy of Tropical Agricultural Sciences, Haikou 571101, China; xyl951016@163.com (Y.X.); wjhnsc@163.com (J.W.); lcconghn@163.com (C.L.); zy563696@163.com (Y.Z.); heartone@126.com (L.G.); yangyang@catas.cn (Y.Y.); 2 School of Plant Protection, Hainan University, Haikou 570228, China; 3 College of Life Sciences, Henan Agricultural University, Zhengzhou 450002, China; guangxtian169@163.com

**Keywords:** *Fusarium oxysporum* f. sp. *cubense* race 4 (Foc4), *Bacillus velezensis*, free amino acids, soluble sugar, transcriptome, disease resistance

## Abstract

Banana wilt disease is an important disease in banana production, causing significant losses. Herein, we investigated the mechanism by which *Bacillus velezensis* isolate X5 enhances the resistance of different resistant banana cultivars to *Fusarium oxysporum* f. sp. *cubense* race 4 (Foc4). From the perspectives of metabolism, transcriptome, and key genes in important pathways, this study analyzed the composition and content changes of other types of signaling molecules, such as free amino acids and soluble sugars, in resistant/susceptible varieties. The results indicate that under pathogen stress, the contents of root-secreted metabolite components in both resistant and susceptible varieties increase significantly overall, and the increase in susceptible varieties is generally higher. For example, the free amino acid components in susceptible varieties are significantly more than those in resistant varieties. However, the addition of biocontrol bacteria can inhibit this increase. Exogenous addition experiments prove that differential metabolites can either promote or inhibit Foc4 and X5 at certain concentrations. The results of KEGG (Kyoto Encyclopedia of Genes and Genomes) enrichment and GO (Gene Ontology) annotation show that resistant varieties have more defense pathways compared to susceptible varieties. Under X5 treatment, more defense genes in resistant varieties are activated or their expression is enhanced, promoting the plant roots to secrete more substances related to plant resistance, such as phenylpropanoids and lignin. This research revealed the effects of *Bacillus velezensis* on bananas and pathogens, allowing valuable conclusions to be drawn. The results have good application potential to understand the relationships among the three species, defining the biocontrol effect and mechanism of *Bacillus velezensis*, and providing a theoretical basis for the biological control of soil-borne diseases such as banana wilt disease.

## 1. Introduction

Banana (*Musa nana* Lour.) is an important fruit crop in tropical and subtropical areas. China has cultivated bananas for more than 3000 years [1]. In 2020, China’s banana production ranked second in the world, with banana production and import volumes of 11,872,600 tons and 1,819,200 tons, respectively, accounting for 9.91% and 7.78% of the world’s total production. However, in recent years, banana wilt disease has seriously threatened the sustainable development of the banana industry. Currently, research is focusing on promoting the safe and sustainable development of the banana industry [2]. Banana wilt is a devastating soil-borne disease caused by *Fusarium oxysporum f. sp. cubense* (Foc) infection of the banana vascular bundle [3]. It can spread rapidly through the soil, by disease-carrying buds, rain, and other methods, and is difficult to completely eradicate. Australia was the first country to detect the disease in 1874 [4]. A subsequent outbreak of the disease occurred in Panama in 1927 [5]. Widespread outbreaks of the disease occurred between 1950 and 1959; however, it began to decline with the introduction of the Cavendish banana [6]. However, this decline did not last long, and a new strain (Foc4) subsequently appeared, which not only showed very aggressive pathogenicity, but also infected almost all banana cultivars, including Cavendish. Almost the entire global banana industry has suffered as a result, including most of the banana-growing areas in Guangdong and Hainan provinces [7].

The control of banana *Fusarium* wilt by antagonistic microorganisms and the cultivation of banana varieties with high resistance are current research foci [8]. Studies have shown that a variety of microorganisms, including *Purpureocillium lilacinum*, *Bacillus subtilis*, and *Streptomyces*, have significant antagonistic effects against *Fusarium oxysporum* [9,10,11]. Breeding disease-resistant varieties is considered an ideal approach at present because of its economic suitability [12]. However, the disease-resistance mechanisms of resistant varieties remain mostly unknown. However, it has been determined that the various root exudates secreted by plants are closely related to plant resistance, representing the initial stage of the plant’s resistance to pathogen invasion [13]. Analyses of root secretions have revealed differences and similarities between resistant and susceptible varieties of watermelon, cotton, soybean, and other species. The root secretions of resistant and susceptible varieties had opposite effects on the growth of pathogens causing wilt: The root secretions of resistant varieties mostly inhibited pathogen growth, while the root secretions of susceptible varieties mostly promoted it [14].

Root exudates not only create a special living environment for various microorganisms in the soil rhizosphere, but also provide them with a variety of nutrients [15]. Plants also secrete amino acids, carbohydrates, organic acids, flavonoids, and other compounds into the soil, and various types of fungi and bacteria in the soil are attracted by these root secretions, thus gathering around the rhizosphere [16]. For example, legumes attract the colonization of mycorrhizal fungi through the secretion of flavonoids [17], malic acid promotes the growth of *Bacillus subtilis* [18,19], and citric acid promotes the growth of *Bacillus amyloliquefaciens* strain SQR9 [20]. In the interaction between plants and microorganisms, plants secrete root exudates. These exudates are capable of altering the community structure and functions of rhizosphere microorganisms, exerting an impact on the growth, reproduction, and metabolism of the microorganisms. At the same time, microorganisms respond to these exudates and, in turn, influence the plant’s own physiological processes such as growth, development, and immunity through various means, thus forming a dynamic feedback regulation mechanism. Currently, there have been extensive research reports on the interaction relationship between Bacillus and plants that is based on root exudates. Mwita et al. [21] found that *Bacillus atrophaeus* could activate certain genes regulating life activities in response to factors secreted by maize roots, thus promoting its own growth. Ling et al. [22] found that cinnamic acid, a type of organic acid in root secretions, had a certain toxic effect on the plant itself, and the tested strain of *Bacillus polymyxa* used in their study significantly reduced the content of cinnamic acid, which was speculated to be a way for the strain to promote plant growth. However, other studies have shown that certain factors can inhibit the growth of Fusarium spores on watermelon, such as coumaric acid, indicating the complex role of root exudates between plants and microorganisms [23].

Metabolomics technology, with its powerful analytical capabilities, can conduct accurate qualitative and quantitative analyses of metabolites that perform diverse biological functions in animals and plants. By systematically monitoring the dynamic changes in metabolite levels, this technology can deeply explore the internal relationships between these changes and the physiological alterations experienced by organisms. Starting from the microscopic small—molecule level, it can meticulously analyze the root causes of physiological changes in organisms and actively explore corresponding effective solutions [24]. In addition, transcriptome sequencing uses high-throughput sequencing of a species’ mRNA in a specific environment to explore the changes in its gene expression in response to that environment [25]. Taking pathogen stress as an example, the key genes related to plant resistance can be screened from among genes showing significant changes in expression, and the reason for plant resistance can be further analyzed at the gene level [26]. In addition, Kasote et al. [27] analyzed significant changes in the levels of amino acids, organic acids, and other compounds after pathogen infection of resistant and susceptible watermelon varieties compared with those in control varieties. The authors discovered multiple metabolic compounds, such as melatonin and jasmonate-isoleucine, that can promote plant defense. Through transcriptome sequencing of soybean plants inoculated with arbuscular mycorrhizal fungi, Zhang et al. [28] found that the expression levels of multiple genes related to resistance were upregulated significantly after inoculation, such as those encoding calcium-dependent protein kinase and phenylalanine ammonia-lyase, thus enhancing the ability of soybeans to resist pathogen invasion.

The present study aims to identify the components of banana root exudates and their levels to comprehensively study the interactions among the components of banana root exudates, Foc4, and the *Bacillus velezensis* isolate X5. We also aim to analyze the response of X5 to banana root exudates and the mechanism by which X5 enhances banana’s resistance to Foc4. The composition and content changes in organic acids, free amino acids, and soluble sugars in the root exudates of the susceptible cultivar “Brazilian” (Musa spp. AAA group, BX) and the disease-resistant cultivar “Nantianhuang” (Musa spp. AAA group, NTH) are analyzed under different treatment conditions using high-performance liquid chromatography (HPLC) and gas chromatography–mass spectrometry (GC-MS). An in-depth exploration of the components of root exudates is also conducted, with a focus on identifying the most significantly affected components. On this basis, the impacts of these components with significant differences on the growth processes of X5 and Foc4 are systematically analyzed, and the key action mechanisms of root exudates in the plant–microbe interaction system are deciphered from a microscopic perspective. The root RNA of each treatment group is extracted and transcriptome analysis performed. Certain differentially expressed genes (DEGs) related to plant resistance are selected for fluorescence quantitative PCR analysis to verify the changes in their expression levels. The correlations between the differences in the levels of free amino acids and soluble sugars and the transcriptome are analyzed, and the positive and negative correlations between the differences in the levels of root exudate components and the expression of related regulatory genes are explained based on their interaction. The mechanism of plant–microorganism interaction is revealed from the perspective of molecular plant pathology and physiology, providing a theoretical basis for the biological control of soil-borne diseases, such as banana wilt.

## 2. Materials and Methods

### 2.1. Test Material and Inoculation

The tested strains were *Fusarium oxysporum* f. sp. *cubense* race 4 (Foc4) and *Bacillus velezensis* X5, both of which were isolated and identified by the Laboratory of Microbial Resources Research and Utilization, Institute of Environment and Plant Protection, Chinese Academy of Tropical Agricultural Sciences, Dongfang City, Hainan Province. The banana varieties tested included tissue culture seedlings of the susceptible cultivar BX and the disease-resistant cultivar NTH, both of which were provided by the seedling tissue culture center of the Chinese Academy of Tropical Agricultural Sciences, Danzhou City, Hainan Province.

Yang’s [29] research findings confirm that the method of directly inoculating bacteria onto hydroponic seedlings can be applied to the screening of the pathogenicity of pathogenic bacteria. By using this method, the resistance of different banana germplasm resources to *Fusarium oxysporum* f. sp. *Cubense* race 4 can be evaluated under the conditions set in the laboratory, providing crucial technical support for the in-depth exploration of the interaction mechanism between banana germplasm resources and pathogenic bacteria. On this basis, a total of eight treatments were set up in this study, with six seedlings/hydroponic box as one treatment, and each treatment was entailed three replicates.

The eight treatments comprised the following: (1) Foc4 spore suspension inoculation of plant roots of hydroponically grown BX; (2) X5 bacterial suspension inoculated on the roots of hydroponically cultured BX; (3) Foc4 spore and X5 bacterial suspensions simultaneously inoculated into the roots of hydroponically cultured BX; (4) Foc4 spore suspension inoculation of plant roots of hydroponically grown NTH; (5) X5 bacterial suspension inoculated on the roots of hydroponic cultured NTH; (6) Foc4 spore and X5 bacterial suspensions simultaneously inoculated into the roots of hydroponic NTH; (7) a control comprising untreated roots of hydroponic BX; and (8) a control comprising untreated roots of hydroponic NTH. The final concentration of the inoculated Foc4 spore suspensions was 1 × 10^6^ cfu/mL when added to the hydroponic solution, and the final concentration of inoculated X5 bacterial suspension was 1 × 10^7^ cfu/mL when added to the hydroponic solution.

### 2.2. Assessment of Infection Symptoms

After inoculation of BX and NTH for 28–30 d, the bulbs of the plants were sliced longitudinally, and the disease incidence on the bulbs was observed according to the browning degree of the corms. The statistical analysis was carried out according to the grading criteria by Mohamed [30].

The formula for calculating the disease index is as follows:

Disease index = ∑ (Number of diseased plants at all levels × Corresponding disease grade) × 100/(Total plant number × Highest disease grade)

Incidence rate (%) = Number of diseased plants/Total plant number × 100%

### 2.3. Collection and Determination of Root Exudates

The collection of root exudates referred to the experimental method of Xu et al. [31], although with some modifications. Banana plants were taken out on the 7th day after inoculation, washed with sterile water two to three times, the liquid in the hydroponic box was replaced with deionized water, and the root secretions were continuously collected for 48 h in an indoor environment with sufficient light. The collected solution was sterilized by passage through a 0.22 μm aqueous microporous filter membrane. The filtered root secretion solution was divided into centrifuge tubes and frozen at −20 °C. After freezing completely, the solution was dried to a powder using a freeze-drying machine at low temperatures and stored for later use.

Referring to the method of Li et al. [32], although with slight modifications, the free amino acid components and contents in the root secretion of bananas collected under different treatments were determined. The freeze-dried powder of the root exudates was dissolved in ultra-pure water as the sample to be measured. Then, 20 μL of the sample to be tested was mixed with 60 μL of AccQ·Fluor boric acid buffer (Waters, Milford, MA, USA) and then with 20 μL AccQ·Fluor derivative reagent (Waters) and vortex mixed. Finally, the mixture was heated in the oven at 55 °C for 10 min to complete the derivative treatment. The proportion of the gradient elution solvent is shown in Supplementary Table S1.

The collected freeze-dried powder of the root exudates was placed into a suitably dry refrigerator and sent to Novogene (Beijing, China) to complete the determination of soluble sugar components and contents in the root exudates. Including machine samples, 26 types of sugar and sugar alcohols were mixed for sample preparation as standards. An Agilent GC-MS apparatus (Santa Clara, CA, USA; 7890A/5975C) was used for detection and analysis, and data in the range of 50 to 600 mass-to-charge ratio (*m*/*z*) were collected in full scanning mode. The original data were processed using Agilent ChemStation workstation software (E.02.02.1431), and the compounds were determined qualitatively and quantitatively.

To determine the aromatic phenolic acids in the root secretions, HPLC was used according to the method of Qu et al. [33], with some modifications. The collected root exudate powder was dissolved in 500 μL of 50% methanol–water solution and then passed through a 0.22 μm organic filter membrane to produce the root exudate phenolic acid detection sample. Ten phenolic acid standards were prepared and tested using HPLC. The HPLC gradient elution procedure is shown in Supplementary Table S2.

### 2.4. Effects of Differentially Abundant Components in Root Exudates on the Growth of X5 and Foc4

The experiment to determine the effect of different amino acids on Foc4 growth was based on the method of Wu et al. [34] with slight modifications. Potato dextrose agar (PDA) solid medium containing valine, alanine, isoleucine, aspartic, glutamic, leucine, methionine, and proline at different concentrations (0, 200, 400, 600, 800 μg/mL) was prepared respectively, and the blank control plates contained no exogenous amino acids.

The pathogen Foc4 was cultured on PDA solid medium for 7 days before the experiment. A 5 mm diameter cake was taken along the edge of the plate colony and inoculated into the center of PDA solid medium containing different concentrations of amino acids, with three repeats for each treatment. After 5 days of incubation at 28 °C, the growth of the Foc4 colony was observed. The colony diameter was calculated by the cross method, and the final colony diameter was calculated by subtracting the 5 mm cake diameter. Each treatment was repeated three times and the whole test was repeated twice.

To determine the effects of different amino acids on the growth of X5, Luria Bertani (LB) liquid medium containing valine, alanine, isoleucine, aspartic acid, glutamic acid, leucine, methionine, and proline at different concentrations (0, 200, 400, 600, 800 μg/mL) was prepared. The blank control group was grown in medium with no exogenous amino acid added. A single X5 colony was selected one day in advance, inoculated in liquid LB, shaken overnight at 37 °C and 180 r/min, and cultured to an OD_600_ of about 1.0. In a sterile environment, the same amount of bacterial suspension was inoculated into LB liquid medium with different concentrations of amino acids, and each treatment was repeated three times. The OD_600_ of each treatment was measured using an ultraviolet spectrophotometer after the culture was shaken at 180 r/min at 37 °C for 12 h. Each treatment was repeated three times and the whole test was repeated twice.

Differentially abundant soluble sugars and organic acids were tested for their effects on the growth of Foc4 and X5, with reference to the method of exogenous addition of differentially abundant amino acids. The differentially abundant soluble sugars were maltose, fructose, galactose, mannitol, glucose, inositol, sucrose, trehalose, and arabinose. The differentially abundant phenolic acid components were cinnamic acid, phthalic acid, and p-hydroxybenzoic acid. In the experiment on the effect of different soluble sugars on Foc4 growth, the medium was changed to base Czapek-Dox Medium. Each treatment was repeated three times and the whole test was repeated twice.

Inhibition ratio (%) = (Control colony diameter − Treated colony diameter)/(Control colony diameter − Control cake diameter) × 100%

### 2.5. Extraction, Detection, Quality Inspection, and Library Construction of Banana Root RNA

The root tissues of each treated banana were clipped after being cultured with deionized water for 48 h. After quick cleaning and other treatments, the roots were immediately immersed in liquid nitrogen. The samples were then removed from the liquid nitrogen, placed in pre-prepared self-sealing bags, frozen again in liquid nitrogen, and then placed in a box containing sufficient dry ice for transport. Shanghai Biotree Biomedical Technology Co., Ltd. (Shanghai, China) was commissioned to complete the RNA extraction, library construction, and Illumina sequencing. The raw data obtained from the sequencing of resistant/susceptible banana varieties were filtered, including the removal of low-quality reads containing Ns and those with linkers. Meanwhile, the GC content, Q30, and Q20 of the clean data were calculated. HISAT2v2.0.5 was used to compare the sequences of the paired-end clean reads with the banana reference genome [35]. The fragments per kilobase of transcript per million mapped reads (FPKMs) of each gene were calculated based on the length of the gene, and the number of reads mapped to that gene was used to estimate the expression level of the gene.

### 2.6. Identification of DEGs and Their Enrichment Analysis

The differential expression analysis between two treatments was performed using DESeq2 (1.16.1) [36]. ClusterProfiler R (3.4.4) was used to perform Gene Ontology (GO) enrichment analysis of the DEGs [37]. A GO term with a corrected *p*-value lower than 0.05 was regarded as significantly enriched for a DEG. ClusterProfiler R was used to analyze the statistical enrichment of DEGs in the Kyoto Encyclopedia of Genes and Genomes (KEGG) pathway analysis.

### 2.7. Reverse Transcription–Quantitative PCR (RT-qPCR) Analysis

Certain genes were selected to verify the transcriptome results using RT-qPCR. A reverse transcription kit from Takara (Dalian, China; RR047A) was used to reverse transcribe isolated RNA to cDNA, which was then used as a template for the qPCR reaction. The PCR amplification instrument was an ETC811 thermocycler (Eastwin, Suzhou, China). The real-time PCR target genes and primers used are shown in Supplementary Table S3. The reaction system was as follows: cDNA template: 1 μL, primers (F + R): 1 μL, 2 × qPCR Mix: 10 μL, plus ddH_2_O to 20 μL. The thermal cycling conditions for qPCR amplification were: predenaturation at 95 °C for 3 min, followed by 40 cycles of 95 °C for 5 s, 60 °C for 5 s, and 72 °C for 15 s.

The experimental data were analyzed and calculated using the ΔΔ cycle threshold (CT) method [38]. The banana actin gene was selected as the internal reference. The Ct value of the target gene in sample 1 was set as CtA1, the Ct value of the reference gene in sample 1 was set as CtB1, the Ct value of the target gene in sample 2 was set as CtA2, and the Ct value of the reference gene in sample 2 was set as CtB2. The expression level of the target gene in sample 2 was 2^−ΔΔCT^ of the expression level of the target gene in sample 1. ΔΔCT = (CtA2 − CtB2) − (CtA1 − CtB1).

### 2.8. Statistical Analysis

Excel (Microsoft, Redmond, WA, USA) and SPSS 26 (IBM Corp., Armonk, NY, USA) were used to process the test data and analyze the significance of the differences (in the figures, different lowercase letters represent significant differences at the 0.05 level). GraphPad Prism 8 (GraphPad Inc., La Jolla, CA, USA) was used for mapping. Origin 2022 (OriginLab Corp., Northampton, MA, USA) was used to map the correlations between the metabolome and transcriptome data. The Pearson correlation coefficient was used for correlation analysis.

## 3. Results

### 3.1. Resistance of BX and NTH to Pathogen Foc4

**Figure 1:**
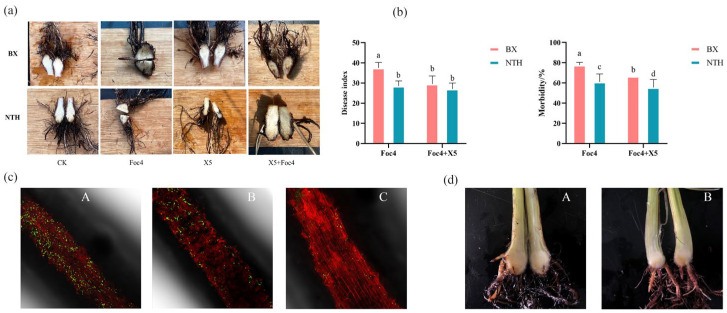
Infection by Fusarium oxysporum in resistant/susceptible banana varieties. (**a**) Corm profile observation of BX and NTH under each treatment. After inoculation treatment, continue the cultivation using the hydroponic method for banana seedlings, with sterile water irrigation as the control. Within 3 days after inoculation, shake on a horizontal shaker at 100 revolutions per minute (r·min^−1^) for 30 min every 24 h to ensure that *Foc4* spores fully contact the roots of banana seedlings. (**b**) Disease index statistics of BX and NTH treated with Foc4 and Foc4 + X5 alone; different lowercase letters indicate the significant difference in the same index (disease index/incidence) at the 0.05 level; the same applies below. (**c**) Colonization of Foc4 spores on the root surface of BX after inoculation for 24 h. A: adding root exudates of BX; B: adding sterile water; C: adding root exudates of NTH. (**d**) Infection degree of Fusarium oxysporum on different resistant plants after 20 d of inoculation under hydroponic conditions. A: BX; B: NTH.

Under hydroponic conditions, compared with the control (CK), the disease symptoms of bananas with different resistances were observed after infection with pathogenic fungi (Figure 1a). No symptoms were found in plants treated with water (CK) and those treated with the biocontrol bacteria X5. According to the results of the investigation (Figure 1b), the overall severity of the disease was as follows: NTH (X5 + Foc4) < NTH (Foc4) < BX (X5 + Foc4) < BX (Foc4), and there were significant differences among the same indicators (disease index/incidence; *p* < 0.05). The results showed that the resistance of disease-resistant cultivar NTH was stronger than that of BX, and the addition of biocontrol bacteria had a significant effect on both of them. In addition, at 24 h after inoculation with Foc4 spores, the root exudates of the resistant variety inhibited the colonization of BX banana roots by the pathogenic spores (Figure 1c). The resistant and susceptible varieties exhibited varying degrees of disease resistance at 20 d after inoculation with the pathogen (Figure 1d). The infection of the pathogen could be observed in the corms of BX, while NTH showed no significant browning.

### 3.2. Identification and Analysis of Free Amino Acids in Root Exudates of Bananas with Different Resistances

The mixture of 17 amino acid standards showed good separation, and the linear correlation coefficient (R2) between the mixed labels of different gradients was greater than 0.99, indicating a good linear relationship. Therefore, the standard curve could be used for the detection and analysis of the experimental samples. The peak time and standard curve values are shown in Supplementary Table S4.

As shown in Supplementary Tables S5 and S6, under Foc4 stress, the levels of free amino acids in the root secretions of the different resistant and susceptible cultivars changed significantly, with the total content of both cultivars being significantly higher than that of CK, and the range of the changes in the susceptible cultivar being significantly greater than that of the resistant cultivar. Sixteen amino acids were detected in BX, while only 11 were detected in NTH. Under Foc4 treatment, the total amino acid content in BX was the highest, reaching 2.44 mg/100 mL. Compared with the treatment of CK, the total amino acid content in BX treated with Foc4 is 1.82 times that in BX treated with CK. Glutamine, valine, isoleucine, cysteine, and alanine levels increased by 2.64, 2.14, 2.5, 3, and 7.25 times, respectively, in BX compared with CK. In the treatment with Foc4 inoculation, the total amount of amino acids secreted by NTH is approximately 1.51 times that secreted in the CK treatment, and threonine was not detected; however, the secretion of histidine and methionine increased, and the valine, cystine, tyrosine, and phenylalanine levels increased by 6.71, 1.17, 1.86, and 2.00 times, respectively. Under treatment with X5 alone, 14 amino acids were detected in NTH and 15 were detected in BX; however, the total amino acid content of NTH was 1.56 times higher than that of BX. Under Foc4 + X5 treatment, the composition and content of free amino acids in BX and NTH were lower than those under CK treatment. No phenylalanine, aspartic acid, histidine, isoleucine, arginine, methionine, or leucine were detected in the BX exudate. Only nine amino acids were detected in the NTH exudate, and the total amino acid secretion was close to that of CK. Further analysis showed that under Foc4 + X5 treatment, only glutamic acid was uniformly present in BX and was significantly increased compared with that in CK, while only alanine and valine had a significant interaction. In NTH, only methionine and glutamic acid were present and were significantly increased compared with that in CK, and only glutamic acid was significant with respect to each other.

### 3.3. Identification and Analysis of Soluble Sugars in Root Exudates of Different Resistant Bananas

The 26 types of sugar and sugar alcohols were well separated, and the linear correlation coefficient (R2) between the mixed labels of different gradients reached more than 0.99, indicating a good linear relationship; thus the standard curve could be used for the detection and analysis of the experimental samples. The peak time and standard curve data are shown in Supplementary Table S7.

As shown in Supplementary Tables S8 and S9, a total of 21 soluble sugar components were detected in BX and NTH. The sucrose content was the highest among them; however, no xylitol, galactitol, 5-ketogluconic acid, lactose, or turanose were detected in CK. The total soluble sugar content of BX under CK treatment was 1.23 times that of NTH. Under Foc4 stress, the soluble sugar content in the root exudates of both groups changed significantly. Sucrose, which accounted for the largest proportion, increased significantly in the BFoc4 group (BX treated with Foc4), but decreased significantly in the NFoc4 group (NTH treated with Foc4). In BX, fructose, mannitol, galactose, inositol, sucrose, arabinose, maltose, trehalose, and glucose levels increased by 2.44, 1.35, 5.36, 5.96, 2.60, 2.41, 2.73, 2.95, and 2.36 times, respectively. The total content of soluble sugars was the highest under treatment with Foc4, at up to 1495.52 ng/mL, being 2.56 times that of CK. In NTH, arabinose, fructose, galactose, glucose, mannitol, and trehalose levels increased by 2.68, 2.57, 1.96, 2.32, 3.64, and 1.86 times, respectively; however, the sucrose content decreased, indicating a 66.3% inhibition of its secretion compared with that of CK. Inositol and galactose levels increased the most in BX, and mannitol increased the most in NTH. Under X5 treatment, the increase in each component in BX was significantly lower than that in the Foc4 treatment group, such as galactose, fructose, glucose and sucrose, whose levels increased by 1.22, 1.76, 1.19, and 1.36 times, respectively, and the total content increased by 1.40 times. In NTH, arabinose, fructose, mannitol, trehalose, glucose, and sucrose levels increased by 1.45, 3.22, 4.24, 2.36, 0.74, and 0.88 times, respectively, and the overall content was about 94.97% of that of CK. Under Foc4 + X5 treatment, the overall soluble sugar content of BX was lower than that of CK (69.33%). In NTH, the sucrose component, with the largest proportion, was close to that of CK under Foc4 + X5 treatment. Further analysis showed that under the two treatments (X5 and Foc4 + X5), mannitol was the most increased sugar in BX and NTH: NFoc4 + X5 (4.37 times) > NX5 (4.24 times) > BX5 (3.64 times) > BFoc4 + X5 (3.10 times). This suggested that mannitol might play an important role in plant pathogen stress.

### 3.4. Identification and Analysis of Organic Acids in Root Exudates of Bananas with Different Resistances

The mixture of 10 phenolic acid standards showed good separation and had a very good linear relationship in the detection range, with the linear correlation coefficient (R2) reaching more than 0.99. Therefore, the standard curve could effectively identify the types and content of phenolic acids in the root exudates. The peak times and the standard curve data are shown in Supplementary Table S10.

As shown in Supplementary Tables S11 and S12, there were significant differences in the types of phenolic acids in the root secretions from untreated resistant and susceptible bananas. Coumaric acid, phthalic acid, syringic acid, and benzoic acid could be detected in the root secretions of NTH, among which the content of phthalic acid was the highest, whereas none of the 10 phenolic acids were detected in BX (Supplementary Table S11). The overall comparison of phenolic acid secretion under inoculation showed that the types and contents of phenolic acid in root secretions of BX were significantly lower than those of NTH under Foc4 and Foc4 + X5 treatments. In the X5 treatment group, there was little difference in the content of phenolic acids in the root secretions of BX compared with those of NTH; however, more types of phenolic acids were detected in the root secretions of BX than in NTH. Under Foc4 treatment, the secretion of p-hydroxybenzoic acid by NTH increased significantly, and under the same treatment, the content of phthalate in the root exudates of NTH was higher than that of BX. In all treatments, the secretion of cinnamic acid could be detected in the root secretions of NTH inoculated with Foc4 and X5. The content of cinnamic acid in the treatment group inoculated with Foc4 was 35.64 times higher that inoculated with X5.

### 3.5. Effect of the Exogenous Addition of Differential Metabolites on the Growth of Foc4 and X5

Through comprehensive analysis of the determination of free amino acids, soluble sugars, and organic acids in the root exudates of BX and NTH under each treatment, representative differentially abundant metabolites were selected for exogenous addition testing. These included aspartic acid, isoleucine, leucine (none of which was present in the NTH-treated samples, but were present in the BX samples), methionine (not detected in any of the CK treatments of BX and NTH but present in the inoculation treatments), glutamic acid (a component that was present in high levels under the CK treatments in BX and NTH), valine, alanine (the highest secreted component in BX and NTH under Foc4 treatment, respectively), and proline (not detected under the Foc4 treatment). The contents of arabinose, fructose, galactose, glucose, mannitol, inositol, sucrose, maltose, and trehalose were all greater than 1 ng/mL, and the changes were more than 2.3 times after inoculation. For the organic acids, phthalic acid, p-hydroxybenzoic acid, and cinnamic acid showed the most significant differences between treatments.

**Figure 2:**
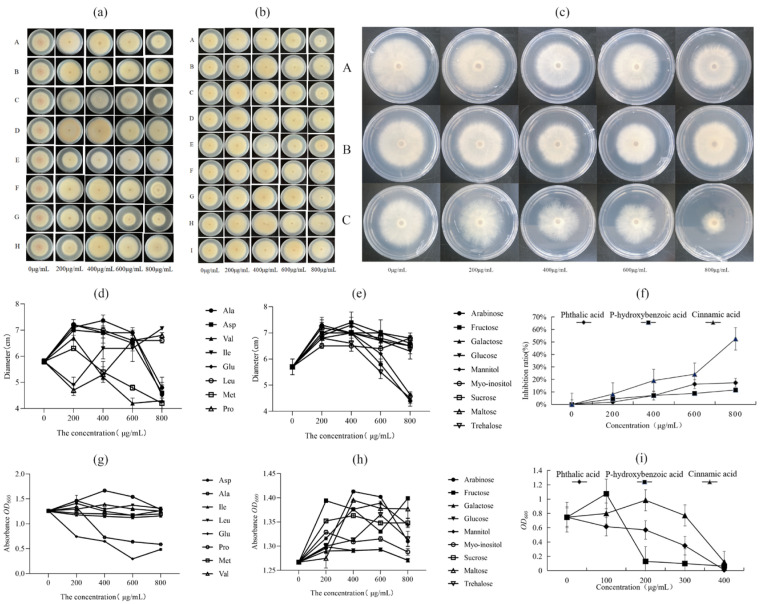
Effect of the exogenous addition of significantly different metabolites on the growth of Foc4 and X5. (**a**) Effect of adding exogenous amino acids on the growth of Foc4. A: alanine; B: glutamic acid; C: methionine; D: leucine; E: proline; F: aspartic acid; G: valine; H: isoleucine. (**b**) Effect of adding exogenous soluble sugars on the growth of Foc4. A: arabinose; B: galactose; C: mannitol; D: fructose; E: trehalose; F: inositol; G: maltose; H: glucose; I: sucrose. (**c**) Effects of exogenous phenolic acids on the growth of Foc4. A: phthalic; B: p-hydroxybenzoic; C: cinnamic. (**d**) Colony diameter of Foc4 on PDA plates containing different concentrations of exogenous amino acids. (**e**) Colony diameter of Foc4 on a plate containing different concentrations of exogenous soluble sugars. (**f**) Inhibition efficiency of three different phenolic acids on the colony diameter of Foc4 on PDA. (**g**) OD600 absorption value of X5 cultured with different amino acids. (**h**) OD600 absorption value of X5 cultured with different exogenous soluble sugars. (**i**) OD600 absorption value of X5 cultured with different exogenous phenolic acids.

As shown in Figure 2, proline, leucine, glutamic acid, and isoleucine promoted Foc4 growth. Alanine, aspartic acid, methionine, and valine at high concentrations (800 μg/mL) inhibited the growth of Foc4, while aspartic acid and valine also promoted the growth of X5. Fructose, galactose, glucose, inositol, sucrose, and maltose showed growth-promoting effects on Foc4 and X5 at different concentrations, among which sucrose and fructose had the most significant growth-promoting effects on X5. At 400–800 μg/mL, arabinose, trehalose, and mannitol significantly inhibited the growth of Foc4, with the highest inhibition rates of 22.81, 21.05, and 17.54%, respectively; moreover, they promoted the growth of X5. The concentrations of the three phenolic acids correlated positively with the growth inhibition rate of Foc4. Among them, cinnamic acid had the strongest inhibitory effect, reaching 52.52% inhibition at 800 μg/mL. In addition, cinnamic acid also had an inhibitory effect on the growth of X5, reaching 98.66% at 400 μg/mL. The inhibitory rates of phthalic acid and p-hydroxybenzoic acid on Foc4 were relatively low, at 17.30% and 11.54% at 800 μg/mL, respectively. By contrast, they had the highest growth-promoting effect on X5 at 100 μg/mL and 200 μg/mL, respectively.

### 3.6. Sequencing and Analysis of the Root Transcriptome of BX and NTH Under Each Treatment

In this experiment, four treatments were carried out on resistant and susceptible banana varieties, namely CK, Foc4 alone, X5 alone, Foc4 + X5, and each treatment was repeated three times. Transcriptome sequencing was performed on 24 samples after total RNA processing. After filtering, the original sequencing amount, the effective sequencing amount, the Q20%, the GC (guanine and cytosine) content, and the Q30% were determined, as shown in Supplementary Table S13. The total information content of each sample of BX and NTH was about 6G, and the retention ratio of effective reads was greater than 95%. The Q20% was higher than 99%, the Q30% was higher than 97%, and the GC content was higher than 47.5%, indicating high data quality after pretreatment. To obtain read information, Hisat2 was used to compare the reference genome with the pre-processed valid data. As shown in Supplementary Tables S13 and S14, the number of reads that could be matched to the genome among the 24 samples ranged from 74.80% to 93.13%.

**Figure 3:**
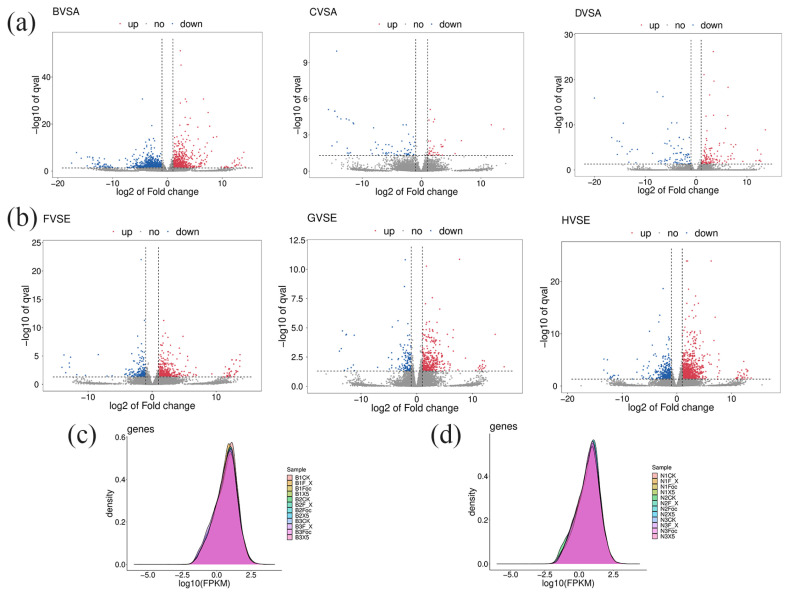
Transcriptome analysis of BX and NTH under different treatments. (**a**) Statistical diagram of differentially expressed genes in M. acuminata AAA Cavendish cv. Baxi under different treatments compared to the water control. Panel B vs. A shows the differentially expressed genes compared to CK treated with Foc4 alone; panel C vs. A shows the differentially expressed genes compared to CK treated with X5 alone; panel D vs. A shows the differentially expressed genes between the Foc4 + X5 treatment and CK. (**b**) Statistical diagram of differentially expressed genes between different treatments of NTH and the water control; panel F vs. E shows the differentially expressed genes compared to CK treated with Foc4 alone; panel G vs. E shows the differentially expressed genes compared to CK treated with X5 alone; panel H vs. E shows the differentially expressed genes between the Foc4 + X5 treatment and CK. (**c**) FPKM analysis of genes from three replicates of different treatments in M. acuminata AAA Cavendish cv. Baxi, BCK: the distribution map of gene expression under water control; BFoc: distribution map of gene expression after treatment with Foc4 alone; BX5: distribution map of gene expression after single biocontrol treatment; BF_ X: gene expression levels under cotreatment of biocontrol bacteria and Foc4. (**d**) Analysis of FPKM values for three replicates of different treatments of NTH. NCK: distribution map of gene expression under the water control; NFoc: distribution map of gene expression after treatment with Foc4 alone; NX5: distribution map of gene expression after a single biocontrol treatment; NF_X: gene expression levels under cotreatment of biocontrol bacteria and Foc4.

Based on the Hisat2 comparison results, Stringtie was used to reconstruct transcripts and to calculate the expression levels of all genes in each sample (as the FPKM value). Gene expression levels in different samples were determined, and the density distribution map of gene expression values in all samples was drawn according to the FPKM value. The horizontal coordinate was log_10_(FPKMs), and the vertical coordinate was gene density. The expression distribution of the 24 samples was approximately normal, and the statistical results for the expression levels in each group were basically similar; however, the density peaks of each group were different, indicating that although the overall expression trend of the samples was similar, there are still obvious differences (Figure 3c,d).

### 3.7. Transcriptomic Analysis and Functional Analysis of DEGs

**Figure 4:**
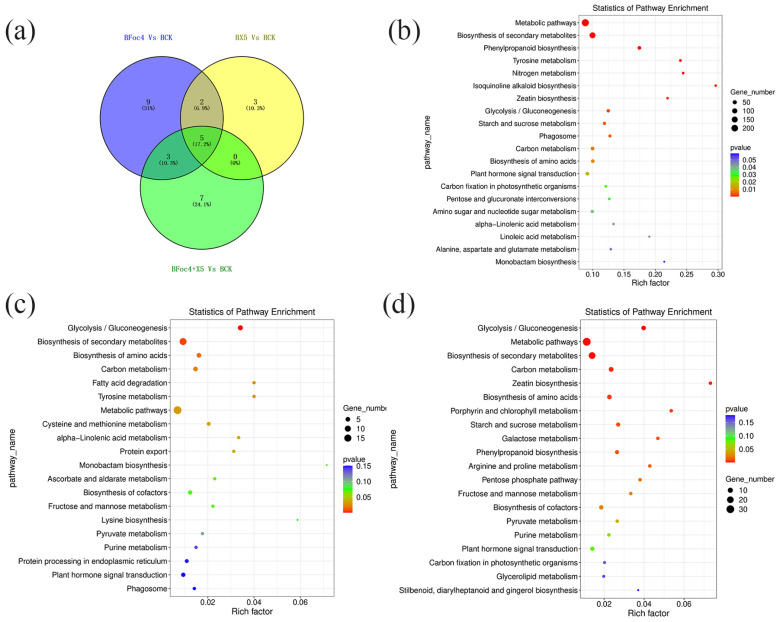
Transcriptome analysis of BX under different treatments. (**a**) Venn diagram of KEGG pathways with significant differences under different treatments of M. acuminata AAA Cavendish cv. Baxi. (**b**) KEGG functional classification of the root transcriptome of M. acuminata AAA Cavendish cv. Baxi under Foc4 treatment compared with CK. (**c**) KEGG functional classification of the root transcriptome of M. acuminata AAA Cavendish cv. Baxi under single plus X5 treatment compared with CK. (**d**) KEGG functional classification of the root transcriptome of M. acuminata AAA Cavendish cv. Baxi under Foc4 + X5 treatment compared with CK.

**Figure 5:**
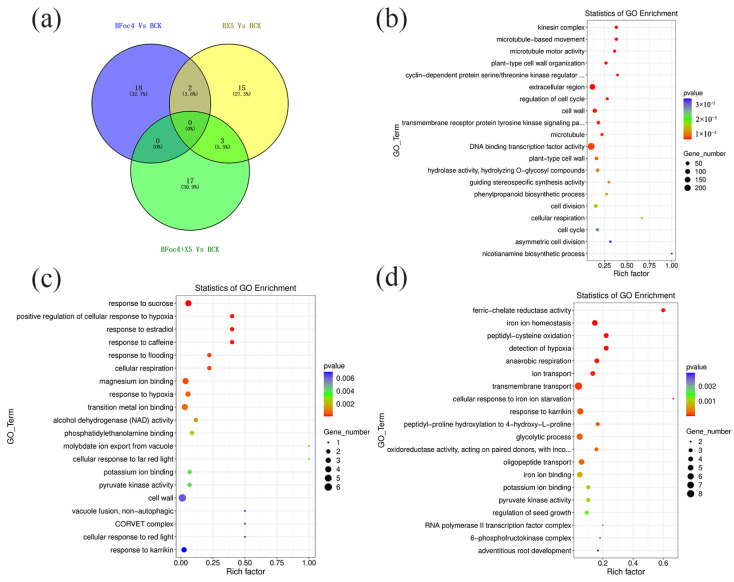
Transcriptome analysis of BX under different treatments. (**a**) GO annotation Venn diagram of significant differences under different treatments of M. acuminata AAA Cavendish cv. Baxi. (**b**) GO annotation classification of the root transcriptome of M. acuminata AAA Cavendish cv. Baxi under single Foc4 treatment compared with CK. (**c**) GO annotation classification of the root transcriptome of M. acuminata AAA Cavendish cv. Baxi under single X5 treatment compared with CK. (**d**) GO annotation classification of the root transcriptome of M. acuminata AAA Cavendish cv. Baxi under Foc4 + X5 treatment compared with CK.

**Figure 6:**
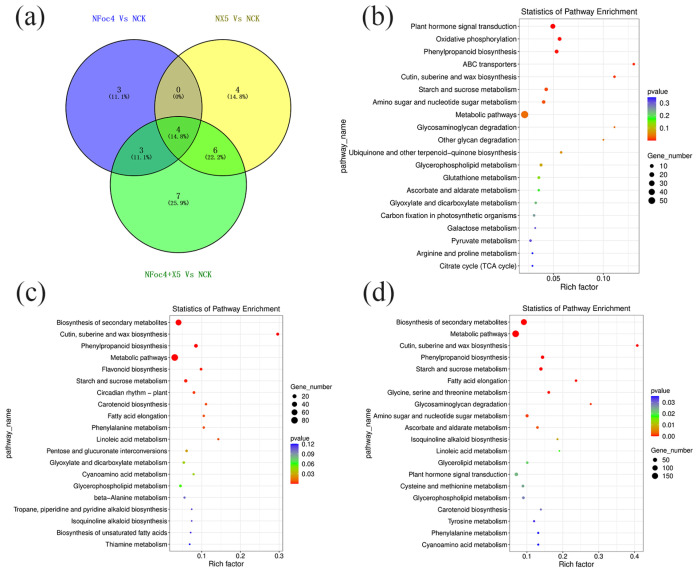
Transcriptome analysis of NTH under different treatments. (**a**) Venn diagram of KEGG pathways with significant differences under different treatments of NTH. (**b**) Classification of KEGG functions of the root transcriptome of NTH under Foc4 treatment compared with CK. (**c**) Classification of KEGG functions of the root transcriptome of NTH under single plus X5 treatment compared with CK. (**d**) Classification of KEGG functions of the root transcriptome of NTH under Foc4 + X5 treatment compared with CK.

**Figure 7:**
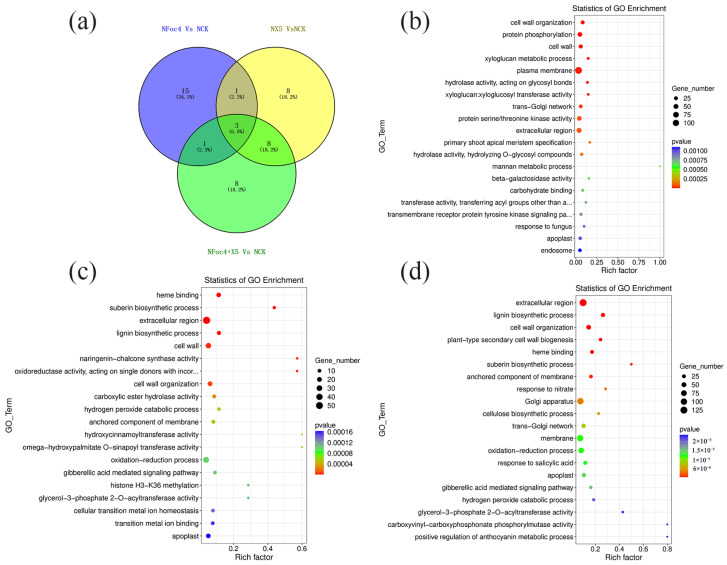
Transcriptome analysis of NTH under different treatments. (**a**) Venn diagram of the GO annotation of significant differences under different treatments of NTH. (**b**) GO annotation classification of the root transcriptome of NTH under single Foc4 treatment compared with CK. (**c**) GO annotation classification of the root transcriptome of NTH under single X5 treatment compared with CK. (**d**) GO annotation classification of the root transcriptome of NTH under Foc4 + X5 treatment compared with CK.

In accordance with the requirement of satisfying both |log_2_ fold-change (FC)| ≥ 1 and q < 0.05, a total of 2352 DEGs were identified in BX under treatment with Foc4 alone, comprising 1028 upregulated genes and 1324 downregulated genes. By contrast, 691 DEGs were identified in NTH under treatment with Foc4 alone, with 493 upregulated genes and 198 downregulated genes. A total of 104 DEGs were identified in BX under X5 treatment only, with 37 upregulated genes and 67 downregulated genes. In NTH under X5 treatment only, 586 DEGs were identified, with 440 upregulated genes and 146 downregulated genes. Under Foc4 + X5 treatment, 209 DEGs were identified in BX, with 127 upregulated genes and 82 downregulated genes, whereas in NTH, 1463 DEGs were identified, with 999 upregulated genes and 464 downregulated genes. With log_2_FC as the horizontal coordinate and -log_10_ (qvalue) as the vertical coordinate, volcano maps were drawn for all genes in the differential expression analysis (Figure 3a,b). KEGG and GO enrichment analyses were performed for the DEGs in each treatment group of BX and NTH compared with CK, and the top 20 KEGG pathways and GO terms with the smallest *p* values were selected, respectively, to draw bubble maps (Figure 4, Figure 5, Figure 6 and Figure 7).

Five KEGG pathways were coenriched significantly under each treatment in BX (Figure 4a): Metabolic pathways (ko01100), Biosynthesis of secondary metabolites (ko01110), Glycolysis/Gluconeogenesis (ko00010), Carbon metabolism (ko01200), and Biosynthesis of amino acids (ko01230). Glycolysis/Gluconeogenesis and Carbon metabolism were downregulated under each treatment. Among the associated DEGs, LOC103973721 encodes pyruvate decarboxylase, and was significantly downregulated only in the BFoc4 group, but was not in the BX5 and BFoc4 + X5 groups. Biosynthesis of amino acids was upregulated in the BFoc4 group and downregulated in the BX5 and BFoc4 + X5 groups. Further analysis showed that X5 treatment downregulated the expression levels of the LOC103982456 and LOC103993455, and BFoc4 + X5 treatment downregulated the expression levels of LOC103978680, LOC103980923, and LOC103998788. LOC103982456, LOC103978680, and LOC103998788 all of which encode proteins that regulate the expression of pyruvate kinase. In addition, we found that LOC104000682, encoding acetyl lactate synthase, was significantly downregulated in the BFoc4 group, but not in the BX5 and BFoc4 + X5 groups. There was no coenriched KEGG pathway in the BX5 and BX5 + Foc4 treatment groups. Three KEGG pathways were coenriched in the BFoc4 and BX5 + Foc4 groups, namely Zeatin biosynthesis (ko00908), Starch and sucrose metabolism (ko00500), and Phenylpropanoid biosynthesis (ko00940). Zeatin biosynthesis was significantly upregulated in both the BFoc4 and BX5 + Foc4 groups, whereas the other two pathways were downregulated in the BFoc4 group and upregulated the BX5 + Foc4 group. In Starch and sucrose metabolism, in the BX5 + Foc4 group, treatment specifically upregulated the expression of LOC103999830, which encodes a protein that regulates the expression of trehalose 6-phosphate phosphatase. LOC103970589 and LOC103977324, which encode proteins controlling phenylpropanoid biosynthesis, were significantly expressed in the BX5 + Foc4 group. LOC103970589 encodes a protein that regulates the expression of peroxidase, and LOC103977324 encodes a protein that regulates the expression of trans-cinnamic acid 4-monooxygenase; the former was upregulated and the latter was downregulated.

In BX, under each treatment, only three GO Terms were coannotated between the BX5 and BFoc4 + X5 groups (Figure 5a), which were response to Karrikin (GO:0080167), potassium ion binding (GO:0030955), and pyruvate kinase activity (GO:0004743). The response to Karrikin was upregulated, whereas the remaining two were downregulated. LOC103969348 and LOC103995115, which encode proteins that regulate the response to Karrikin, were significantly upregulated in both the BX5 and BFoc4 + X5 groups. The former gene encodes the transcription factor HY5 and the latter encodes an early photoinducible protein. Interestingly, further analysis found that several GO items related to iron ions were significantly upregulated in the BFoc4 + X5 group, including iron homeostasis, ion transport, cellular response to iron ions, iron chelate reductase activity, and iron ion binding. LOC103993181 encodes iron reductive oxidase, which is involved in the regulation of iron ion homeostasis, ion transport, iron chelate reductase activity, and iron ion binding. LOC103995331 encodes the transcription factor bHLH10, which is involved in the regulation of iron ion homeostasis and cell reaction to iron ions. Both genes were upregulated in the BFoc4 + X5 group.

There were four KEGG pathways coenriched significantly under each treatment of NTH (Figure 6a), namely, Metabolic pathway (ko01100), Starch and sucrose metabolism (ko00500), Phenylpropanoid biosynthesis (ko00940), and Cutin, suberine and wax biosynthesis (ko00073), with each pathway being significantly upregulated. Associated with starch and sucrose metabolism, LOC103984186, encoding endoglucanase, was significantly upregulated in the NFoc4 group, but showed no significant change in the NX5 and NX5 + Foc4 groups. Phenylpropanoid biosynthesis was promoted in the NFoc4 group and downregulated in the BFoc4 group, which verified that NTH has certain Foc4 resistance properties compared with BX, and mounts a more active defense against pathogen infestation. In NTH, 10 genes that regulate the phenylpropanoid biosynthesis pathway were identified, of which only LOC103991820 was common to both BX and NTH, suggesting that each has its own defense mechanism against Foc4 invasion. Three KEGG pathways, Glycosaminoglycan degradation (ko00531), Amino sugar and nucleotide sugar metabolism (ko00520), and Plant hormone signal transduction (ko04075), were coenriched in the NFoc4 and NX5 + Foc4 groups, and all three pathways showed significant upregulation under both treatments. Foc4 treatment in NTH regulates aminosugar and nucleotide sugar metabolism differentially via eight genes, whereas 20 genes were regulated in the NX5 + Foc4 group, and the regulatory gene common to both was LOC103973924, encoding UDP-glucuronide 4-epimerase 6-like. In addition, LOC103995656, encoding chitinase, a member of the aminosugar and ribose metabolism pathway that is closely related to plant disease resistance, was significantly upregulated in the NX5 + Foc4 group, with no significant change in the NFoc4 group. Consequently, we hypothesized that X5 induces the rapid expression of LOC103995656 in NTH under stimulation by Foc4. Six KEGG pathways were coenriched in the NX5 and NX5 + Foc4 groups, including biosynthesis of secondary metabolites (ko01110), phenylalanine metabolism (ko00360), linoleic acid metabolism (ko00591), fatty acid elongation (ko00062), glyoxylic acid and dicarboxylic acid metabolism (ko00630), and carotenoid biosynthesis (ko00906). Except for carotenoid biosynthesis, these metabolic pathways showed consistent upregulation. Carotenoid biosynthesis was downregulated under X5 treatment but upregulated under X5 + Foc4 treatment. Carotenoids can absorb and transmit light energy and protect chlorophyll, playing an important role in improving plant immunity [39].

There were three GO terms (Figure 7a) jointly annotated by NTH under each treatment, namely extracellular region (GO:0005576), cell wall tissue (GO:0071555) and apoplast (GO:0048046), all of which were significantly upregulated. The GO Term coannotated in the NFoc4 and NFoc4 + X5 groups was extracellular region (GO:0005576), which was significantly upregulated in both groups. The NX5 and NFoc4 + X5 groups had eight GO terms in common: suberin biosynthetic process (GO:0010345), lignin biosynthetic process (GO:0009809), hydrogen peroxide catabolic process (GO:0042744), oxidation-reduction process (GO:0055114), gibberellic acid mediated signaling pathway (GO:0009740), heme binding (GO:0020037), glycerol-3-phosphate2-O-acyltransferase activity (GO:0090447), and anchored component of membrane (GO:0031225) [40,41,42]. Most of these GO entries are related to plant resistance.

**Figure 8:**
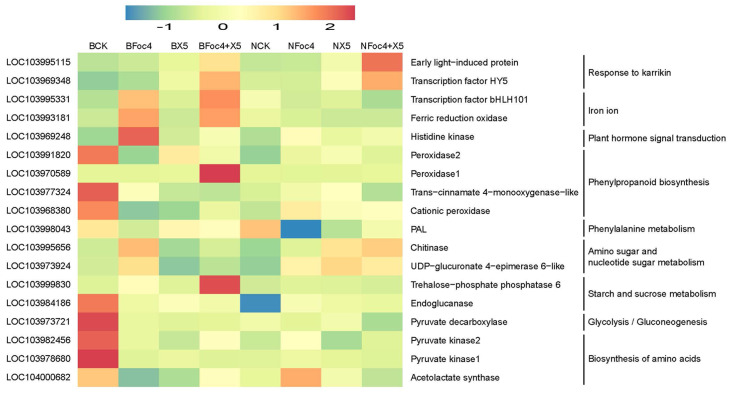
Heat maps showing differentially expressed genes related to plant resistance under each treatment of BX and NTH. Red indicates an increase; green indicates a decrease. BCK: CK treatment of BX; BFoc4: BX inoculated with Foc4; BX5: BX inoculated with X5; BFoc4 + X5: Foc4 and X5 simultaneously inoculated onto BX; NCK: CK treatment of NTH; NFoc4: NTH inoculated with Foc4; NX5: NTH inoculated with X5; NFoc4 + X5: Foc4 and X5 simultaneously inoculated onto NTH.

As shown in Figure 8, the expression of genes related to the regulation of plant resistance in BX and NTH was different in the different varieties and under different treatments. Further KEGG analysis showed that cysteine and methionine metabolism, starch and sucrose metabolism, linoleic acid metabolism, and plant hormone signal transduction were the involved pathways of BX and NTH. Among them, cysteine and methionine metabolism appeared in the BX5 and NX5 + Foc4 groups, both related to X5, and were upregulated. The experiment using exogenous amino acid addition in this study proved that methionine had a significant inhibitory effect on the growth of *Fusarium oxysporum*, which had previously been shown to play an important role in the biological process of fungi, bacteria, and plants [43]. LOC103998043, encoding phenylalanine aminolyase (PAL), was downregulated in both cultivars under pathogen stress. LOC103968380, encoding cationic peroxidase, was downregulated in all treatments of BX, but upregulated in all treatments of NTH. LOC103969248 encodes histidine kinase, which is involved in the regulation of plant hormone signaling, was significantly upregulated only under BFoc4 treatment, while there was no significant change in the other treatments. There was no common GO term for BX and NTH under each treatment, suggesting that both varieties had their own response system to pathogen infection. There were more items related to plant resistance in NTH than in BX, most of which were related to promoting resistance. Moreover, after pathogen infection, the entries related to plant resistance in BX mostly showed inhibitory effects, indicating that NTH has stronger resistance than BX to banana wilt.

### 3.8. Correlation Analysis of the Transcriptomic and Metabolomic Data

**Figure 9:**
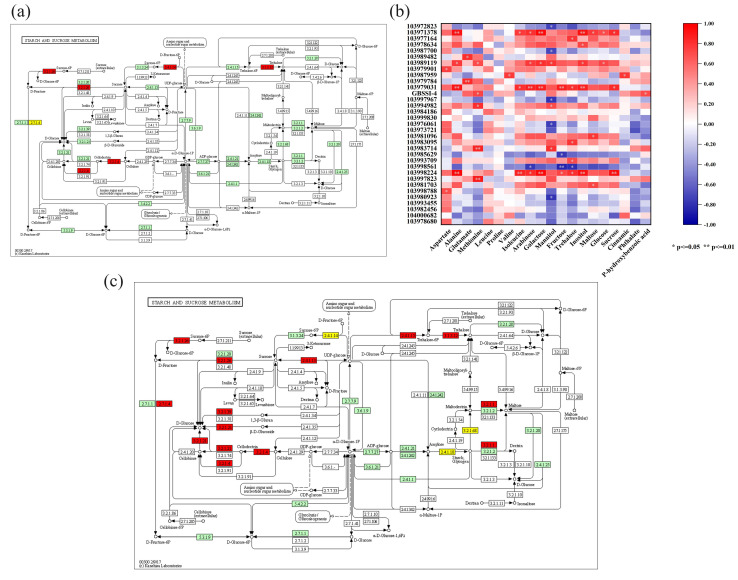
Correlation analysis between differentially abundant metabolites and the transcriptome results. (**a**) Pathways of differentially expressed genes involved in starch and sucrose metabolism under BX5 + Foc4 treatment. Note: red represents an upregulated gene and its encoded protein, yellow represents a downregulated gene and its encoded protein, the same below. (**b**) Analysis of the correlation between differentially abundant metabolites and related differentially expressed genes in different treatments of BX and NTH. (**c**) Pathways of differentially expressed genes involved in starch and sucrose metabolism under NX5 + Foc4 treatment.

**Figure 10:**
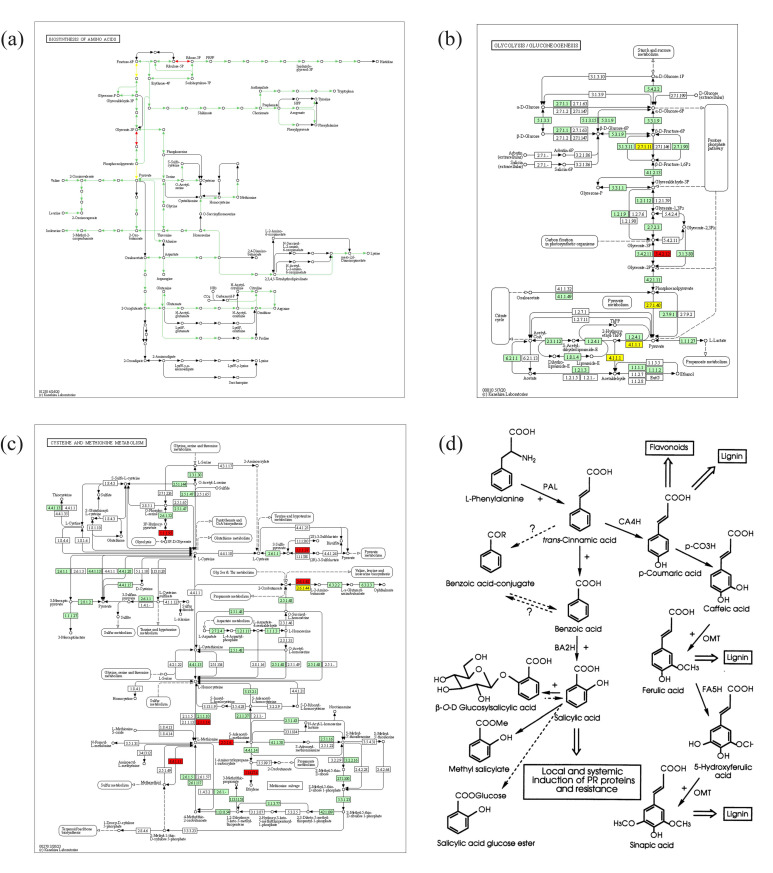
Correlation analysis between differentially abundant metabolites and the transcriptome results. (**a**) Differentially expressed genes involved in the amino acid biosynthesis pathways under BX5 + Foc4 treatment. (**b**) Differentially expressed genes involved in glycolysis/gluconeogenesis metabolism pathways under BX5 + Foc4 treatment. (**c**) Differentially expressed genes involved in cysteine and methionine metabolism pathways under NX5 + Foc4 treatment. (**d**) Schematic diagram of the biosynthetic and metabolic pathways of SA and phenylpropyl. Note: cited from Lee et al. [44].

The differentially abundant metabolites were selected for correlation analysis with the DEGs involved in regulating their formation under each treatment in the BX and NTH samples (Figure 9 and Figure 10). In the figure, red represents a positive correlation, blue represents a negative correlation, and the depth of the color is proportional to the strength of the correlation. As can be seen from Figure 6a, a single gene might correlate with multiple metabolites, and similarly, a single metabolite might correlate with multiple genes, indicating a complex regulatory network between the two. For example, LOC103998788, encoding pyruvate kinase, correlated significantly and positively with aspartic acid and with leucine to a certain extent, which is consistent with the transcriptome sequencing results. In addition, LOC103979031, encoding glucosidase, correlated significantly and positively with multiple soluble sugar components (e.g., arabinose, galactose, fructose, and trehalose), which also indicated that the transcriptome sequencing results correlated well with the metabolome results.

KEGG pathway analysis of the differential abundant metabolites with associated DEGs further demonstrated gene regulation of metabolite synthesis. For example, Figure 6d shows the DEG regulation diagram of amino acid biosynthesis under BX5 + Foc4 treatment. There are seven DEGs involved in the regulation of this pathway, two of which are upregulated and five that are downregulated. The DEGs respond to pathogens by affecting the synthesis of several amino acids, including aspartic acid, valine, and leucine. The results showed that BX and NTH could be protected against harmful invasions by influencing related gene expression at the molecular level under different inoculation treatments.

### 3.9. RT-qPCR Verification of the Transcriptome Data

**Figure 11:**
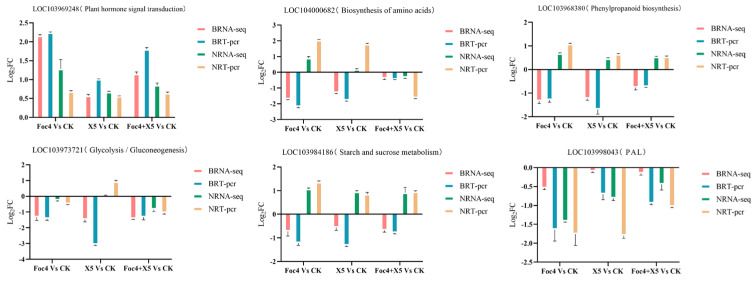
Transcriptome sequencing data and quantitative PCR results for BX and NTH compared with the water control. BRNA-seq: Sequencing value of this gene in BX; NRNA-seq: Sequencing value of this gene in NTH; BRT-pcr: RT-qPCR results of this gene in BX; NRT-pcr: RT-qPCR results of this gene in NTH.

The expression levels of six genes encoding proteins related to plant resistance regulation from the BX and NTH samples were verified using RT-qPCR: pyruvate decarboxylase (LOC103973721), acetyl lactate synthase (LOC104000682), cationic peroxidase (LOC103968380), phenylalanine ammonia lyase (LOC103998043), histidine kinase (LOC103969248), and endoglucanase (LOC103984186). The results showed that the expression trends of the selected genes in the treatment and control groups were generally consistent with the transcriptomic sequencing results (Figure 11). It also indicated that the sequencing results were highly reliable, ensuring the accuracy of the transcriptome data analysis.

## 4. Discussion

Plants contain a large number of metabolites, which not only maintain the growth and development of plants and control various life activities, but also regulate their response to the external environment, including enhancing plant disease resistance and resisting the invasion of pathogenic fungi. Under exposure to diseases, pests, high temperatures, and other environments, plants will regulate their life activities by producing a large number of metabolites, such as amino acids, soluble sugars, and organic acids, allowing them to survive under adverse conditions. Jaiswal et al. [45] and Vora et al. [46] found that by studying the changes in the content and components of metabolites in plants under different living environments, they could infer the changed metabolic pathways, allowing them to make favorable choices for plants.

In this study, HPLC and GC-MS were used to determine the composition and content of free amino acids, organic acids, and soluble sugars in BX and NTH under each treatment. Under CK treatment, there was a significantly greater variety of the types of free amino acids in BX (16 types) than in NTH (10 types), and their content was 1.68 times higher. Under CK treatment, the composition and content of free amino acids in NTH were similar to the results of Gan et al. [47]. A total of 17 amino acids were detected in BX and 14 were detected in NTH. As essential factors for microbial growth [48], the type and content of amino acids in BX were significantly higher than those in the resistant variety. Li et al. [49] found that alanine, glutamic acid, aspartate, phenylalanine, tyrosine, and valine had significant stimulating effects on the growth of spores of pathogenic fungi. Marschner [50] showed that plants were extremely susceptible to disease when glutamic acid and alanine were present in high concentrations. In this study, except for aspartate, leucine, and isoleucine, which were detected only in BX, the other amino acids were found in BX and NTH. There was a greater increase in amino acids in BX under Foc4 stress than in the resistant variety, possibly creating a more favorable growth environment for the pathogenic fungi. This prompted us to speculate that this was one of the reasons behind their susceptibility to wilt disease. However, after the addition of X5, the overall amino acid secretion of BX and NTH became close to that of the control. It was speculated that under the combined environment of Foc4 and X5, the biocontrol bacteria either inhibited or killed the pathogenic fungi via antagonism, or they reduced the levels of amino acids through competition [51], such that the amino acid levels were significantly lower than those when Foc4 and X5 were administered separately. The exogenous addition test (Figure 2a) showed that isoleucine, leucine, and aspartate were only detected in the susceptible variety BX; however, these three amino acids were not detected when BX was treated with X5 + Foc4, further proving that BX was more susceptible to the growth and invasion of pathogenic fungi than NTH. Methionine was not detected in CK treatment of BX and NTH; however, the secretion of methionine was detected under Foc4 and X5 treatment. The content of valine increased significantly when BX and NTH were infected by Foc4, but was close to that of the control group after the addition of X5. We speculated that the biocontrol bacteria achieved the control effect by competing with or inducing BX to reduce the secretion of these amino acids.

As the main carbon source for microbial growth, soluble sugars provide nutrition and energy. Regarding soluble sugars in root exudates, most current studies only measured the total sugar content or only measured common sugars, such as fructose, sucrose, and glucose. In our study, the differences in the composition and content of soluble sugars in BX and NTH were further determined. We detected 21 soluble sugars, and those related to disease resistance were further explored. Sucrose was the most abundant sugar in BX and NTH. Under CK treatment, the total soluble sugar content and the contents of fructose, glucose, and sucrose in BX were higher than those in the resistant variety. The content of carbohydrates in root exudates of BX was significantly higher than that of the resistant variety. Under Foc4 stress, the levels of various soluble sugars changed significantly in BX and NTH, and the increase in BX was larger than that in the resistant variety, which also led to a higher sugar environment in BX compared with that in NTH. Usman et al. [52] found that *Fusarium oxysporum*, as a pathogenic fungus with a higher nutrient requirement for carbon and a higher nitrogen ratio, was more likely to cause pathogen stress in a high-sugar environment. Therefore, we speculated that this might be one of the factors that made BX more susceptible to wilt disease. When X5 was added, the levels of soluble components in BX and NTH were close to those of the control, which was consistent with the change trend of amino acids under the same treatments. The exogenous addition test (Figure 2b) showed that arabinose and mannitol, which inhibit Foc4, showed higher increases in the resistant variety than in BX, which prompted us to hypothesize that this pattern is a strategy to inhibit the growth of the pathogen in NTH. When treated with X5 + Foc4, sucrose levels decreased in BX and increased in the resistant variety. We speculated that X5 and Foc4 competed for the utilization of sucrose in BX because of the reduced resistance of the plants.

Sivaram et al. [53] showed that low molecular weight organic acids in many root exudates and residual matter after decomposition play a central role in plant roots’ influence on the microflora structure. Li et al. [54] found that the contents of coumaric acid, benzoic acid, and total phenolic acid in the root secretions of cultivars resistant to peanut blight were much higher than those in susceptible cultivars. The results of the present study showed that the content and types of phenolic acids in the root secretion of the resistant variety were significantly higher than those in BX, which was consistent with previous studies on the relationship between *Fusarium* wilt resistance and the phenolic acid content in root secretions. The abundance and diversity of phenolic acids in the root secretions of the resistant variety were significantly higher than those in the root secretions of BX under CK treatment. Foc4 treatment significantly increased the variety and content of phenolic acids in the root exudates of BX and NTH, and the increase in the resistant variety was significantly greater than that of BX, among which cinnamic acid, p-hydroxybenzoic acid, and phthalic acid showed the most obvious differences. Treatment with X5 increased the type and content of phenolic acids in the root exudates of BX and NTH, making the type and amount of phenolic acids in the root exudates of BX similar to that of NTH, which might be one of the mechanisms by which X5 improves the resistance of BX to banana wilt. The results of the exogenous addition experiment (Figure 2c) showed that cinnamic acid, p-hydroxybenzoic acid, and phthalic acid had certain inhibitory effects on the mycelial growth of pathogenic fungi, and the inhibitory effects were as follows: cinnamic > phthalic > p-hydroxybenzoic. Cinnamic acid could only be detected in root exudates of disease-resistant plants inoculated with Foc4 or X5, which suggested that the secretion of cinnamic acid by the roots of disease-resistant plants plays a certain role in promoting plant resistance to pathogen infection.

The transcriptome sequencing results showed that the expression levels of 2352 genes in BX showed significant changes under Foc4 stress, while only 691 genes in the resistant variety showed significant changes, indicating that the life activities of BX were more active under pathogen stress, resulting in (or resulting from) increased gene expression. The much lower number of DEGs in NTH than in BX might be related to its resistance to *Fusarium oxysporum*, which is similar to the results reported by Kaushal et al. [55]. According to free amino acid and soluble sugar determination results, the secretion of both components in BX under pathogen stress was significantly higher than that in NTH, which might be related to the lower number of DEGs in NTH.

Analysis of the DEGs between BX and NTH relative to CK under pathogen stress showed that most of the genes related to defense enzymes and their regulatory pathways were significantly downregulated in BX. For example, pyruvate decarboxylase (LOC103973721) and acetyl lactate synthase (LOC104000682) were significantly downregulated only in BX. Downregulated expression of pyruvate decarboxylase made plants more susceptible to disease [56], while the authors also reported that downregulated expression of acetyl lactate synthase inhibited the synthesis of valine, which has a significant inhibitory effect on pathogens. In our study, the determination of free amino acids showed that the content of valine in BX increased significantly under Foc4 treatment, suggesting that Foc4 might induce the downregulation of these two genes to create an environment conducive to pathogen invasion. The results of RT-qPCR further supported this speculation. LOC103998043 (encoding PAL) and phenylpropanoid biosynthesis were also downregulated, both of which are related to plant resistance [57]. PAL is involved in the synthesis of plant resistance factors, such as phytoalexins and lignin, which are important for the plant stress response [58]. As shown in Figure 10d, PAL catalyzes the cleavage of L-phenylalanine into cinnamic acid [44]. Inhibition of this enzyme would inhibit the formation of cinnamic acid, which is a fungicidal organic acid that acts against Foc4. The RT-qPCR results (Figure 7) showed that X5 increased PAL expression, thus alleviating the inhibition of cinnamic acid formation. We speculated that this is one of the ways by which X5 enhances plant resistance. In addition, LOC103969248 (encoding histidine kinase) was significantly upregulated only in BX. A study by Terrettaz et al. [59] showed that histidine kinase plays an important role in plant stress. Combined with our results, these observations suggest that upregulation of histidine kinase might be a method by which BX resists pathogen invasion. In the resistant variety, most of the genes related to defense enzymes and their regulatory pathways were significantly upregulated, such as phenylpropanoid biosynthesis and plant hormone conduction. The disease-resistant variety could induce the upregulation of endoglucanase (LOC103984186) and UDP-glucuronic 4-epimerase 6-like (LOC103973924) under Foc4 stimulation. The former enhances plant self-defense, and the latter can promote the synthesis of glycoproteins and glycolipids in the downstream pathway. Glycoproteins are used for cell recognition and cell communication, and glycolipids are important components of cell surface antigens [60], which are speculated to be involved in resistance to pathogen invasion in the resistant variety.

The methods by which X5 enhanced resistance to Foc4 in BX and NTH were significantly different, with more methods being employed in BX. When BX was stimulated by Foc4, X5 further upregulated the expression of trehalose 6-phosphate phosphatase (LOC103999830), peroxidase (LOC103970589), iron reduction oxidase (LOC103993181), and transcription factor bHLH101 (LOC103995331). Trehalose 6-phosphate phosphatase can not only activate the expression of multiple stress-responsive genes [61], but also causes the accumulation of trehalose and glucose in the downstream regulatory pathway. This result was similar to the change in the soluble sugar content under BX5 + Foc4 treatment measured in this study. An exogenous soluble sugar test also proved that trehalose could significantly inhibit the growth of fungi, and peroxidase can use the corresponding single-chain alcohol compounds to synthesize lignin [62] to enhance plant resistance. Iron reduction oxidase not only supports improved plant resistance, but also plays an important role in the reduction of iron ions and the regulation of transport [63]. The transcription factor bHLH101 can promote the absorption and transport of iron ions in plants. Sun et al. [64] found that *Bacillus* showed a particularly high ability to produce ferriferous carriers in the process of antagonizing *Fusarium oxysporum* infection. The strain used in this study, X5, was proven to produce iron carriers in our laboratory; therefore, we speculated that X5 might inhibit the activity of *Fusarium oxysporum* by promoting the absorption and transport of iron ions in bananas. X5 also downregulated the expression of LOC103978680 and LOC103998788 (encoding pyruvate kinase) and LOC103977324 (encoding trans-cinnamic acid 4-monooxygenase). Pyruvate kinase can inhibit the process of amino acid biosynthesis [65], which correlates with the determination of free amino acids under BX5 + Foc4 treatment. Moreover, inhibition of the expression of trans-cinnamic acid 4-monooxygenase can restrict the catalytic transformation of trans-cinnamic acid into other compounds, resulting in the accumulation of trans-cinnamic acid, which plays an important role in plant resistance to pathogen stress [66]. In the resistant variety, X5 further upregulated the expression of the genes encoding 9-cis epoxycarotenoid dioxygenase (NCED) (LOC103990944), zeaxanthin 7,8(7′,8′)-lytic dioxygenase (LOC103982044), and chitinase (LOC103995656). Zeaxanthin 7,8(7′,8′)-lytic dioxygenase and NCED are both involved in abscisic acid (ABA) biosynthesis, in which NCED is considered to be a key enzyme. NCED could improve the resistance of wild *Arabidopsis thaliana* to drought stress [67], suggesting that it might also play a role in the resistance of NTH to Foc4 stress. Chitinase is closely related to plant resistance [68] and can directly act on the fungal cell wall to degrade it, thus exerting an antifungal role. In addition, X5 also promotes the formation of many factors related to plant resistance, such as hematoxylin, cutin, suberine, and lignin. The biosynthetic pathways of these molecules were not significantly enriched in the susceptible variety under the same treatment. Moreover, X5 could promote the metabolism of sucrose, which has a significant promoting effect on plant growth, and methionine, which has a significant inhibiting effect on the growth of pathogenic fungal spores, which might represent one way by which X5 enhances Foc4 resistance of NTH.

Based on the composition and content changes of free amino acids, soluble sugars, and organic acids in the root exudates of BX and NTH under different treatments and the transcriptome sequencing results, this study provided several suggested mechanisms by which *Bacillus velezensis* enhances the resistance of BX and NTH to Foc4. The results of our study provide a powerful theoretical basis for the biological control of soil-borne diseases, such as banana wilt. However, the experiments in this study were conducted under hydroponic conditions; therefore, there are still some limitations considering the more complex environment faced by bananas in actual agricultural production. Therefore, the application of X5 in the field is worthy of further research.

## Data Availability

All RNA-seq data have been uploaded to the SRA (Sequence Read Archive) of NCBI with accession number PRJNA1100744. This BioProject accession number is provided instead of SRP to facilitate searching in Entrez.

## Supplementary Materials

The following supporting information can be downloaded at: https://www.mdpi.com/article/10.3390/jof11050379/s1, Table S1. The proportion of the gradient elution solvent. Table S2. Phenolic acid HPLC gradient elution procedure. Table S3. Primer sequence used in real-time fluorescence quantitative PCR. Table S4. Peak sequences, peak times, and regression equations of phenolic acid standards. Table S5. Types and contents of amino acids in the BX root exudates of bananas under different treatments (mg/100 mL). Table S6. Types and contents of amino acids in the root exudates of NTH under different treatments (mg/100 mL). Table S7. Peak sequences, peak times, and regression equations of sugar and sugar alcohol standards. Table S8. Types and contents of soluble sugars in the root exudates of BX under different treatments (ng/mL). Table S9. Type and content of soluble sugars in the root exudates of NTH under different treatments (ng/mL). Table S10. Peak sequences, peak times, and regression equations of phenolic acid standards. Table S11. Types and contents of organic acids in root exudates of BX under different treatments (μg/Plant). Table S12. Types and contents of organic acids in root exudates of NTH under different treatments (μg/Plant). Table S13. Statistics of the Root Sequencing Data of BX and NTH under different treatments. Table S14. Comparison of Reference Genes in the Root Sequencing Data of BX and NTH under different treatments.
