# Supplementary material for: Bacillus velezensis Isolate X5 Stimulates the Resistance of Resistant and Susceptible Banana Varieties to Foc Through Different Mechanisms
Source: J Fungi (Basel). 2025 May 16;11(5):379. doi: 10.3390/jof11050379 (PMC12113440; doi:10.3390/jof11050379)
Supplement: Supplementary file 1 [file jof-11-00379-s001.zip › jof-3492972-supplementary.pdf]

Table S1. The proportion of the gradient elution solvent

Time / AB	0 min	0.5 min	15 min	19 min	32 min	33 min	34 min	37 min	38 min	50 min
A/%	100	98	93	90	67	67	0	0	100	100
B/%	0	2	7	10	33	33	100	100	0	0

Table S2. Phenolic acid HPLC gradient elution procedure

Time (min)	Acetonitrile	2% Acetic acid-Water
0	5%	95%
16	22%	78%
18	10%	90%
23	29%	71%
30	40%	60%
42	10%	90%
45	5%	95%

Table S3. Primer sequence used in real-time fluorescence quantitative PCR

Primers	Primer sequence	Regulatory pathway
9248-F	ATGGTGGAACTGGTATTGGCTTGAG	Plant hormone signal
9248-R	AGGACCGCAGTGAATGTGAATGTG	transduction
0682-F	CTGACCTCTTGCTCGCACTTGG	Biosynthesis of amino
0682-R	GGCTGCTTGTTCTTCCCGATCTC	acids

8380-F	GGTGCGTCGGAGTAGGAAGAGG	Phenylpropanoid
8380-R	GAAGTGGAGGAGAGGAAGACAATGC	biosynthesis
3721-F	GGAGAGCAAGCCAGTGTATATCAGC	Glycolysis /
3721-R	TTGGGCGTGAGAAAGTAAGGAATCG	Gluconeogenesis
4186-F	TGTTGTGGGATTTGGTCAGCGATAC	Starch and sucrose
4186-R	TCCCTCCACTTCCATCCTCCTTTG	metabolism
8043-F	GCTGAAGCGTGTCGGAATCGG	PAL
8043-R	GGCTAGGATGTTGGCGTCGTAAC	

Table S4. Peak sequences, peak times, and regression equations of phenolic acid standards

Peak sequence	Peak time	Type of amino acid	Regression equation	R^2
1	6.055	Asp	y = 846995.9570 x - 6725.1667	0.9975
2	7.045	Glu	y = 922022.1935 x - 7521.2500	0.9969
3	12.512	Ser	y = 1109343.4839 x - 8748.6250	0.9969
4	13.397	Gly	y = 2519085.2473 x - 19114.7917	0.9972
5	14.493	His	y = 1653694.9677 x - 12743.2500	0.9971
6	16.554	Arg	y = 1401190.0215 x - 10993.5417	0.9972
7	17.398	Thr	y = 2262973.4194 x - 18006.3750	0.9974
8	18.316	Ala	y = 1380226.4086 x - 11427.9167	0.9972
9	19.307	Pro	y = 5516627.2688 x - 43430.8333	0.9972

10	25.859	Tyr	y = 2823325.5914 x - 21790.8333	0.9968
11	26.905	Val	y = 1887557.8495 x - 15228.5833	0.9965
12	27.633	Met	y = 1844352.3441 x - 14187.4167	0.9972
13	28.312	Cys	y = 1166064.1720 x - 5332.4167	0.9927
14	29.684	Ile	y = 2382333.4194 x - 12992.8750	0.9959
15	30.008	Leu	y = 2098702.7957 x - 8323.9167	0.9920
16	31.529	Phe	y = 2541354.3226 x - 17473.2500	0.9983
17	32.977	Lys	y = 2076274.2366 x - 13702.3333	0.9920

Table S5. Types and contents of amino acids in the BX root exudates of bananas under different treatments (mg/100mL)

Amino acid \ Treatments	CK	Foc4	X5	Foc4+X5
Asp	0.15 ± 0.04^{a}	0.16 ± 0.03^{a}	—	—
Glu	0.11 ± 0.02^{c}	0.29 ± 0.08^{a}	0.17 ± 0.01^{b}	0.31 ± 0.09^{a}
Ser	0.13 ± 0.02^{b}	0.15 ± 0.03^{a}	0.13 ± 0.04^{b}	0.13 ± 0.01^{b}
Gly	0.08 ± 0.01^{a}	0.07 ± 0.02^{a}	0.07 ± 0.02^{a}	0.04 ± 0.00^{b}
His	0.06 ± 0.01^{a}	0.06 ± 0.00^{a}	0.07 ± 0.00^{a}	—
Arg	0.07 ± 0.04^{b}	0.10 ± 0.01^{ab}	0.09 ± 0.03^{a}	—
Thr	0.06 ± 0.00^{bc}	0.11 ± 0.02^{a}	0.07 ± 0.01^{b}	0.05 ± 0.00^{c}
Ala	0.08 ± 0.01^{c}	0.58 ± 0.05^{a}	0.11 ± 0.03^{b}	0.07 ± 0.01^{c}
Pro	0.05 ± 0.01^{a}	—	0.05 ± 0.02^{a}	0.05 ± 0.00^{a}

Tyr	0.08 ± 0.02[a]	0.10 ± 0.01[a]	0.09 ± 0.03[a]	0.09 ± 0.00[a]
Val	0.07 ± 0.01[c]	0.15 ± 0.04[a]	0.11 ± 0.03[b]	0.07 ± 0.01[c]
Met	—	0.06 ± 0.00[a]	0.06 ± 0.00[a]	—
Cys	0.06 ± 0.11[b]	0.18 ± 0.04[a]	—	0.06 ± 0.00[b]
Ile	0.04 ± 0.01[b]	0.10 ± 0.00[a]	0.04 ± 0.01[b]	—
Leu	0.05 ± 0.02[a]	0.05 ± 0.00[a]	0.05 ± 0.12[a]	—
Phe	0.06 ± 0.02[b]	0.09 ± 0.01[a]	0.07 ± 0.02[ab]	—
Lys	0.19 ± 0.08[a]	0.20 ± 0.04[a]	0.10 ± 0.10[b]	0.21 ± 0.05[a]
Total content	1.34 ± 0.19[b]	2.44 ± 0.07[a]	1.29 ± 0.22[b]	1.00 ± 0.05[d]

Note: Different lowercase letters in the same row indicate significant differences at the 0.05 level.

Table S6. Types and contents of amino acids in the root exudates of NTH under different treatments (mg/100mL)

Treatments / Amino acid	CK	Foc4	X5	Foc4+X5
Asp	—	—	—	—
Glu	0.19 ± 0.08[c]	0.07 ± 0.01[d]	1.01 ± 0.29[a]	0.25 ± 0.10[b]
Ser	0.06 ± 0.01[a]	0.05 ± 0.00[a]	0.07 ± 0.02[a]	0.06 ± 0.00[a]
Gly	0.04 ± 0.00[a]	0.03 ± 0.00[a]	0.05 ± 0.01[a]	0.04 ± 0.00[a]
His	—	0.09 ± 0.01[a]	0.07 ± 0.00[a]	—
Arg	—	—	0.07 ± 0.00[a]	—

Thr	0.05 ± 0.00^{a}	—	0.05 ± 0.00^{a}	—
Ala	0.05 ± 0.01^{a}	0.05 ± 0.01^{a}	0.06 ± 0.00^{a}	0.05 ± 0.01^{a}
Pro	—	—	0.08 ± 0.03^{a}	—
Tyr	0.07 ± 0.01^{b}	0.13 ± 0.05^{a}	0.08 ± 0.03^{b}	0.08 ± 0.00^{b}
Val	0.07 ± 0.01^{b}	0.47 ± 0.18^{a}	0.07 ± 0.10^{b}	0.06 ± 0.01^{b}
Met	—	0.06 ± 0.00^{a}	0.07 ± 0.01^{a}	0.07 ± 0.01^{a}
Cys	0.06 ± 0.00^{a}	0.07 ± 0.00^{a}	0.07 ± 0.01^{a}	0.07 ± 0.00^{a}
Ile	—	—	—	—
Leu	—	—	—	—
Phe	0.07 ± 0.01^{b}	0.14 ± 0.05^{a}	0.07 ± 0.04^{b}	0.06 ± 0.00^{b}
Lys	0.15 ± 0.10^{b}	0.05 ± 0.00^{c}	0.19 ± 0.11^{a}	—
Total content	0.80 ± 0.09^{c}	1.21 ± 0.09^{b}	2.01 ± 0.16^{a}	0.74 ± 0.05^{c}

Note: Different lowercase letters in the same row indicate significant differences at the 0.05 level.

Table S7. Peak sequences, peak times, and regression equations of sugar and sugar alcohol standards

Peak sequence	Peak time	Type of soluble sugars	Regression equation	R^2
1	16.06	Lyxose	y = 0.08060 x + 0.00165	0.99991
2	16.33	Arabinose	y = 0.15127 x + 0.00898	0.99968
3	16.61	Ribose	y = 0.09357 x - 0.01597	0.9996

4	17.04	Xylitol	y = 0.03964 x + 0.00047	0.99984
5	17.2	Rhamnose	y = 0.12573 x - 0.01154	0.99843
6	17.25	Arabitol	y = 0.03805 x + 0.00632	0.99832
7	17.32	Fucose	y = 0.03194 x + 0.00293	0.99949
8	18.07	Fucitol	y = 0.00899 x + 0.00003	0.99993
9	18.59	D-pinitol	y = 0.01838 x - 0.00154	0.99948
10	19.02	Fructose	y = 0.01119 x + 0.00170	0.99942
11	19.17	Mannose	y = 0.01375 x + 0.00250	0.99864
12	19.21	Galactose	y = 0.01824 x - 0.03195	0.99532
13	19.21	2-Ketogulonic	y = 0.00854 x - 0.05232	0.99393
14	19.27	Glucose	y = 0.00292 x + 0.00013	0.99987
15	19.57	Mannitol	y = 0.02248 x - 0.00021	0.99993
16	19.62	Glucuronic	y = 0.02388 x - 0.03294	0.99621
17	19.63	Sorbitol	y = 0.01812 x + 0.00045	0.99966
18	19.67	Dulcitol	y = 0.00953 x + 0.00104	0.99689
19	19.71	5-Ketogluconic	y = 0.00060 x + 0.00015	0.99924
20	20.17	Gluconic	y = 0.00522 x - 0.00019	0.9999
21	20.88	Myo-inositol	y = 0.04039 x - 0.04700	0.99768
22	24.08	Sucrose	y = 0.00021 x + 0.00109	0.99141
23	24.34	Lactose	y = 0.01140 x - 0.00213	0.99323
24	24.59	Maltose	y = 0.01820 x + 0.00242	0.99747
25	24.6	Trehalose	y = 0.00238 x - 0.00002	0.99813

26	24.62	Turanose	y = 0.00194 x + 0.00015	0.99847

Table S8. Types and contents of soluble sugars in the root exudates of BX under different treatments (ng/mL)

Treatments / Sugar	CK	Foc4	X5	Foc4+X5
Lyxose	0.25 ± 0.02^{c}	0.39 ± 0.09^{a}	0.30 ± 0.05^{b}	0.21 ± 0.02^{d}
Arabinose	1.75 ± 0.36^{c}	4.22 ± 0.43^{a}	2.28 ± 0.94^{b}	2.27 ± 0.07^{b}
Ribose	2.43 ± 0.03^{c}	2.43 ± 0.33^{c}	2.52 ± 0.02^{b}	2.66 ± 0.06^{a}
Xylitol	—	—	—	—
Rhamnose	0.43 ± 0.07^{d}	0.79 ± 0.20^{b}	0.84 ± 0.23^{a}	0.68 ± 0.09^{c}
Arabitol	1.68 ± 0.55^{c}	1.67 ± 0.13^{c}	2.45 ± 0.54^{a}	2.09 ± 0.79^{b}
Fucose	0.24 ± 0.15^{d}	0.51 ± 0.07^{b}	0.54 ± 0.15^{a}	0.26 ± 0.03^{c}
Fucitol	0.20 ± 0.11^{b}	0.26 ± 0.11^{a}	0.16 ± 0.10^{c}	0.15 ± 0.16^{c}
D-pinitol	0.14 ± 0.00^{c}	0.63 ± 0.02^{a}	0.17 ± 0.01^{bc}	0.19 ± 0.04^{b}
Fructose	8.91 ± 0.39^{d}	21.71 ± 1.24^{a}	15.68 ± 0.88^{b}	10.57 ± 0.05^{c}
Mannose	3.02 ± 0.60^{d}	4.08 ± 0.93^{c}	4.23 ± 0.96^{a}	4.12 ± 0.82^{b}
Galactose	2.26 ± 0.04^{d}	12.11 ± 0.50^{a}	2.75 ± 0.23^{c}	2.99 ± 0.50^{b}
2-Ketogulonic	2.46 ± 0.13^{d}	2.72 ± 1.26^{a}	2.63 ± 0.08^{b}	2.55 ± 0.09^{c}
Glucose	8.44 ± 2.34^{d}	19.89 ± 0.92^{a}	10.08 ± 2.22^{b}	9.97 ± 1.13^{c}
Mannitol	2.06 ± 1.28^{d}	9.10 ± 3.24^{a}	7.49 ± 0.66^{b}	6.38 ± 2.18^{c}
Glucuronic acid	0.76 ± 0.36^{a}	—	0.76 ± 0.00^{a}	0.71 ± 0.33^{b}

Sorbitol	0.37 ± 0.06^{c}	0.74 ± 0.01^{a}	0.49 ± 0.04^{b}	0.28 ± 0.03^{d}
Galactitol	—	—	—	—
5-Ketogluconic	—	—	—	—
Gluconic acid	4.29 ± 1.73^{d}	4.81 ± 0.41^{c}	14.21 ± 1.09^{a}	4.91 ± 0.14^{b}
Myo-inositol	1.14 ± 0.17^{b}	6.81 ± 0.05^{a}	0.82 ± 0.03^{d}	0.92 ± 0.01^{c}
Sucrose	535.06 ± 20.29^{c}	1388.60 ± 29.65^{a}	726.54 ± 8.31^{b}	337.26 ± 30.1^{d}
Lactose	—	—	—	—
Maltose	0.63 ± 0.16^{d}	1.71 ± 0.49^{a}	0.83 ± 0.24^{c}	1.38 ± 0.50^{b}
Trehalose	8.22 ± 2.93^{d}	24.24 ± 1.42^{a}	21.92 ± 2.08^{b}	14.84 ± 3.99^{c}
Turanose	—	—	—	—
Total content	584.74 ± 12.3^{c}	1495.52 ± 48.42^{a}	817.70 ± 26.49^{b}	405.39 ± 11.21^{d}

Note: Different lowercase letters in the same row indicate significant differences at the 0.05 level.

Table S9. Type and content of soluble sugars in the root exudates of NTH under different treatments (ng/mL)

Treatments / Sugar	CK	Foc4	X5	Foc4+X5
Lyxose	0.15 ± 0.01^{c}	0.29 ± 0.06^{a}	0.20 ± 0.02^{b}	0.21 ± 0.03^{b}
Arabinose	1.07 ± 0.21^{d}	2.87 ± 0.24^{a}	1.56 ± 0.35^{b}	1.45 ± 0.16^{c}
Ribose	1.47 ± 0.01^{c}	1.80 ± 0.35^{b}	1.13 ± 0.40^{d}	2.83 ± 0.00^{a}
Xylitol	—	—	—	—

Rhamnose	0.31 ± 0.06^{c}	0.62 ± 0.04^{a}	0.46 ± 0.08^{b}	0.29 ± 0.12^{c}
Arabitol	1.37 ± 0.29^{b}	1.82 ± 0.70^{a}	0.94 ± 0.04^{d}	1.25 ± 0.78^{c}
Fucose	0.21 ± 0.10^{d}	0.48 ± 0.05^{a}	0.45 ± 0.09^{b}	0.31 ± 0.06^{c}
Fucitol	0.21 ± 0.03^{c}	0.42 ± 0.02^{a}	0.32 ± 0.05^{b}	0.32 ± 0.03^{b}
D-pinitol	0.10 ± 0.01^{b}	0.18 ± 0.00^{a}	0.12 ± 0.05^{b}	0.11 ± 0.03^{b}
Fructose	5.24 ± 0.05^{c}	13.47 ± 1.81^{b}	16.89 ± 0.10^{a}	2.49 ± 0.08^{d}
Mannose	2.44 ± 0.01^{c}	4.87 ± 0.04^{a}	3.31 ± 0.83^{b}	3.25 ± 0.18^{b}
Galactose	1.57 ± 0.05^{c}	3.08 ± 0.33^{a}	1.44 ± 0.31^{d}	2.31 ± 0.42^{b}
2-Ketogulonic	2.76 ± 0.09^{d}	2.88 ± 1.12^{c}	4.09 ± 0.17^{a}	3.02 ± 0.15^{b}
Glucose	7.05 ± 0.04^{c}	16.37 ± 3.80^{a}	5.20 ± 1.26^{d}	9.55 ± 0.86^{b}
Mannitol	1.75 ± 0.02^{d}	6.37 ± 0.82^{c}	7.42 ± 0.13^{b}	7.64 ± 2.48^{a}
Glucuronic	0.69 ± 0.02^{c}	0.78 ± 0.01^{a}	0.80 ± 0.04^{a}	0.72 ± 0.07^{b}
Sorbitol	0.33 ± 0.01^{d}	1.16 ± 0.07^{b}	0.47 ± 0.09^{c}	1.26 ± 0.03^{a}
Dulcitol	—	—	—	—
5-Ketogluconic	—	—	—	—
Gluconic	2.54 ± 0.10^{d}	5.08 ± 1.45^{b}	4.85 ± 0.14^{c}	5.89 ± 0.37^{a}
Myo-inositol	1.32 ± 0.06^{a}	1.33 ± 0.13^{a}	0.89 ± 0.28^{c}	1.09 ± 0.09^{b}
Sucrose	438.53 ± 9.48^{b}	290.75±38.82^{d}	385.63 ± 8.07^{c}	449.33 ± 4.13^{a}
Lactose	—	—	—	—
Maltose	0.46 ± 0.02^{c}	1.36 ± 0.12^{a}	0.44 ± 0.10^{d}	0.64 ± 0.08^{b}
Trehalose	6.65 ± 4.10^{d}	12.37 ± 0.52^{b}	15.67 ± 0.49^{a}	7.52 ± 0.97^{c}
Turanose	—	—	—	—

Total content	476.23±45.89[b]	368.36±22.36[d]	452.29± 24.89[c]	501.47± 23.54[a]

Note: Different lowercase letters in the same row indicate significant differences at the 0.05 level.

Table S10. Peak sequences, peak times, and regression equations of phenolic acid standards

Peak sequence	Peak time	Phenolic acid	Regression equation	R^2
1	4.736	Gallic	y = 20932x + 56227	0.9906
2	6.796	Coumaric	y = 14083x - 2175.2	0.9969
3	10.216	P-hydroxybenzoic	y = 12813x + 15011	0.9984
4	11.697	Phthalate	y = 4086.4x + 4403	0.9986
5	12.017	Vanillic	y = 14364x + 8471.9	0.9969
6	12.831	Syringic	y = 25723x + 14347	0.9974
7	17.568	Ferulic	y = 28531x + 2031	0.9999
8	20.538	Benzoic	y = 3504x + 694	0.9958
9	22.926	Salicylic	y = 5071x - 1071.4	0.9985
10	30.046	Cinnamic	y = 73587x + 12319	0.9981

Table S11. Types and contents of organic acids in root exudates of BX under different treatments (μg/Plant)

Organic acid \ Treatments	CK	Foc4	X5	Foc4+X5
Gallic	—	0.14 ± 0.03^{a}	—	—
Coumaric	—	0.13 ± 0.07^{a}	0.14 ± 0.06^{a}	—
P-hydroxybenzoic	—	—	0.20 ± 0.02^{a}	—
Phthalate	—	0.72 ± 0.49^{b}	1.53 ± 0.71^{a}	0.65 ± 0.07^{b}
Vanillic	—	—	0.21 ± 0.03^{a}	—
Syringic	—	—	0.12 ± 0.04^{a}	—
Ferulic	—	—	—	—
Benzoic	—	—	1.41 ± 0.65^{a}	—
Salicylic	—	—	—	—
Cinnamic	—	—	—	—
Total content	—	0.98 ± 0.44	3.61 ± 0.08	0.65 ± 0.07

Note: Different lowercase letters in the same row indicate significant differences at the 0.05 level.

Table S12. Types and contents of organic acids in root exudates of NTH under different treatments (μg/Plant)

Organic acid \ Treatments	CK	Foc4	X5	Foc4+X5
Gallic	—	0.27 ± 0.19^{a}	—	—
Coumaric	0.19 ± 0.06^{c}	0.34 ± 0.04^{a}	—	0.27 ± 0.14^{b}
P-hydroxybenzoic	—	0.32 ± 0.12^{a}	0.15 ± 0.04^{c}	0.22 ± 0.05^{b}
Phthalate	0.91 ± 0.69^{d}	2.02 ± 0.64^{a}	1.31 ± 0.46^{b}	1.12 ± 0.35^{c}
Vanillic	—	—	—	—
Syringic	0.04 ± 0.01^{b}	0.02 ± 0.00^{b}	0.08 ± 0.05^{a}	—
Ferulic	—	—	—	—
Benzoic	0.53 ± 0.11^{b}	—	2.00 ± 0.74^{a}	0.65 ± 0.12^{b}
Salicylic	—	—	—	—
Cinnamic	—	0.82 ± 0.25^{a}	0.02 ± 0.00^{b}	—
Total content	1.67 ± 0.72	3.79 ± 0.81	3.57 ± 1.05	2.26 ± 0.54

Note: Different lowercase letters in the same row indicate significant differences at the 0.05 level.

Table S13. Statistics of the Root Sequencing Data of BX and NTH under different treatments

Sample	Raw Data		Valid Data		Valid Ratio (reads)	Q20 %	Q30 %	GC content %
	Read	Base	Read	Base				
B1CK	43311474	6.50G	41882806	6.28G	96.70	99.93	98.11	48.50
B2CK	40051510	6.01G	39030330	5.85G	97.45	99.95	98.35	48.50
B3CK	43805852	6.57G	42316170	6.35G	96.60	99.93	97.96	48.50
B1Foc4	42497616	6.37G	41229534	6.18G	97.02	99.94	98.35	47.50
B2Foc4	39118844	5.87G	38024316	5.70G	97.20	99.95	98.35	48.50
B3Foc4	43127884	6.47G	41766974	6.27G	96.84	99.94	98.15	49.50
B1X5	42903128	6.44G	41034128	6.16G	95.64	99.93	98.27	49.00
B2X5	44145110	6.62G	42728992	6.41G	96.79	99.94	98.29	47.50
B3X5	43735290	6.56G	42369890	6.36G	96.88	99.94	98.05	48.50
B1F+X	39969976	6.00G	38895362	5.83G	97.31	99.96	98.46	49.00
B2F+X	39929160	5.99G	38654910	5.80G	96.81	99.93	98.27	47.50
B3F+X	43715074	6.56G	42515182	6.38G	97.26	99.94	98.14	48.00
N1CK	43594156	6.54G	42042618	6.31G	96.44	99.94	98.07	48.50
N2CK	40246944	6.04G	39100706	5.87G	97.15	99.95	98.28	47.50
N3CK	41610906	6.24G	40085022	6.01G	96.33	99.95	98.51	49.00
N1Foc4	43961028	6.59G	42807704	6.42G	97.38	99.95	98.15	48.50
N2Foc4	39193598	5.88G	37916604	5.69G	96.74	99.95	98.37	47.50

N3Foc4	44482482	6.67G	43075554	6.46G	96.84	99.94	98.31	48.50
N1X5	43380246	6.51G	41915114	6.29G	96.62	99.94	98.06	48.50
N2X5	43852608	6.58G	42142898	6.32G	96.10	99.95	98.30	49.00
N3X5	38937938	5.84G	37641604	5.65G	96.67	99.96	98.38	49.50
N1F+X	38901940	5.84G	37589016	5.64G	96.63	99.93	98.14	49.50
N2F+X	43888284	6.58G	42078742	6.31G	95.88	99.94	98.34	50.00
N3F+X	43553498	6.53G	42087584	6.31G	96.63	99.96	98.45	48.50

Table S14. Comparison of Reference Genes in the Root Sequencing Data of BX and NTH under different treatments

Sample	Valid reads	Mapped reads	Unique Mapped reads	Multi Mapped reads
B1CK	41882806	37291526 (89.04%)	35989844 (85.93%)	1301682 (3.11%)
B2CK	39030330	36119666 (92.54%)	33719327 (86.39%)	2400339 (6.15%)
B3CK	42316170	36003711 (85.08%)	34174657 (80.76%)	1829054 (4.32%)
B1Foc4	41229534	35680027 (86.54%)	33871643 (82.15%)	1808384 (4.39%)
B2Foc4	38024316	33106704 (87.07%)	31073563 (81.72%)	2033141 (5.35%)
B3Foc4	41766974	38418241 (91.98%)	34978042 (83.75%)	3440199 (8.24%)
B1X5	41034128	35789350 (87.22%)	31352503 (76.41%)	4436847 (10.81%)
B2X5	42728992	35316571 (82.65%)	33494082 (78.39%)	1822489 (4.27%)
B3X5	42369890	33337876 (78.68%)	31850438 (75.17%)	1487438 (3.51%)
B1F+X	38895362	34929518 (89.80%)	31344995 (80.59%)	3584523 (9.22%)

B2F+X	38654910	28912116 (74.80%)	27500357 (71.14%)	1411759 (3.65%)
B3F+X	42515182	33803541 (79.51%)	32765870 (77.07%)	1037671 (2.44%)
N1CK	42042618	37665179 (89.59%)	33498634 (79.68%)	4166545 (9.91%)
N2CK	39100706	35365969 (90.45%)	33676030 (86.13%)	1689939 (4.32%)
N3CK	40085022	36552764 (91.19%)	32541001 (81.18%)	4011763 (10.01%)
N1Foc4	42807704	38893821 (90.86%)	35154783 (82.12%)	3739038 (8.73%)
N2Foc4	37916604	33595008 (88.60%)	31172596 (82.21%)	2422412 (6.39%)
N3Foc4	43075554	39593354 (91.92%)	36985758 (85.86%)	2607596 (6.05%)
N1X5	41915114	37114518 (88.55%)	33137954 (79.06%)	3976564 (9.49%)
N2X5	42142898	37902303 (89.94%)	34211531 (81.18%)	3690772 (8.76%)
N3X5	37641604	34910455 (92.74%)	30383149 (80.72%)	4527306 (12.03%)
N1F+X	37589016	34308796 (91.27%)	30088242 (80.05%)	4220554 (11.23%)
N2F+X	42078742	39189881 (93.13%)	32665806 (77.63%)	6524075 (15.50%)
N3F+X	42087584	37932100 (90.13%)	34875848 (82.86%)	3056252 (7.26%)
